# A Framework for Budget-Constrained Zero-Day Cyber Threat Mitigation: A Knowledge-Guided Reinforcement Learning Approach

**DOI:** 10.3390/s26010021

**Published:** 2025-12-19

**Authors:** Mainak Basak, Geon-Yun Shin

**Affiliations:** School of Computer Engineering & Applied Mathematics, Hankyong National University, Anseong-si 17579, Republic of Korea

**Keywords:** Cyber-Threat Knowledge Graph, MITRE ATT&CK, generative cyber range, reinforcement learning for cyber defense, zero-day TTP

## Abstract

Conventional machine-learning-based defenses are unable to generalize well to novel chains of ATT&CK actions. Being inefficient with low telemetry budgets, they are also unable to provide causal explainability and auditing. We propose a knowledge-based cyber-defense framework that integrates ATT&CK constrained model generation, budget-constrained reinforcement learning, and graph-based causal explanation into a single auditable pipeline. The framework formalizes the synthesis of zero-day chains of attacks using a grammar-formalized ATT&CK database and compiles them into the Zeek-aligned witness telemetry. This allows for efficient training of detection using the generated data within limited sensor budgets. The Cyber-Threat Knowledge Graph (CTKG) stores dynamically updated inter-relational semantics between tactics, techniques, hosts, and vulnerabilities. This enhances the decision state using causal relations. The sensor budget policy selects the sensoring and containment decisions within explicit bounds of costs and latency. The inherent defense-provenance features enable a traceable explanation of each generated alarm. Extensive evaluations of the framework using the TTP holdouts of the zero-day instances show remarkable improvements over conventional techniques in terms of low-FPR accuracy, TTD, and calibration.

## 1. Introduction

Modern enterprise’s cyber-defense frameworks encounter three persistent deficiencies. First, it is challenging for detection models to address unseen attack chains that follow the ATT&CK ontology but differ in order, preconditions and tooling [1]. Second, telemetry is not free of charge. The teams operate under tight budgets for log volume, CPU, storage, and latency [2,3]. Third, the explanations are brittle under shifts and are generally not tied to the causal structures of tactics and techniques. Many systems cannot demonstrate which model, which knowledge state, or which configuration produced a given alert [4]. Public datasets and cyber simulation labs help. However, they leave these gaps open. The packet and flow corpora are static in nature. Red team replays are scripted. Most gym environments simulate fixed attack playbooks [2,5]. Logging is usually all-on or fixed per scenario. Causal semantics over ATT&CK are not exposed to the defender or to the evaluation harness. Artifacts such as alerts and explanations are saved as files without verifiable provenance. As a result, we cannot study cost-aware defense, generalization to zero-day chains, or explanation stability in a controlled and reproducible way [6,7,8].

We address these needs with Sim-CTKG, an ATT&CK-aware generative cyber range for reinforcement learning and defense [9]. Nodes represent tactics, techniques, software, hosts, and CVEs [10,11]. Edges encode preconditions and effects that link actions and entities. Sim-CTKG samples unseen technique chains with a formal grammar that respects ATT&CK constraints, then compiles each chain into witness telemetry for network and host logs. The defender observes features and a two-hop CTKG slice each step and learns a sensor-budget policy that chooses both containment actions and which log sources to activate under cost and latency limits [12]. A causal CTKG enables counterfactual probes that quantify preventability and explanation stability on held-out chains [13]. Every alert and explanation ships with a Content Credentials (C2PA) manifest so artifacts are verifiable.

The adversary follows tactic and technique semantics and may vary parameters, tools, and order within those constraints. The defender controls containment actions and logging configuration but cannot modify the attacker [14]. Telemetry is synthesized by the witness compiler and can be calibrated with a small set of real replays when available [15]. The range is not developed as a full digital twin. This is a research-grade environment that isolates the effects of costs, causality, and provenance on learning and detection.

A key design goal of Sim-CTKG is to isolate the effects of three factors that strongly influence real-world cyber-defense systems: (i) cost-aware sensing, (ii) causal structure and counterfactual reasoning, and (iii) verifiable provenance. The environment exposes configuration switches that independently enable or disable budget constraints, CTKG-based causal reasoning, and provenance logging, allowing us to characterize the individual and combined contributions of these components to detection accuracy, generalization, and robustness.

The key contributions of this research are as follows:

1. We develop a grammar-constrained generator for unseen ATT&CK technique chains and a corresponding compiler (Sim-CTKG) that produces structured, Zeek-aligned network and host telemetry consistent with each stage of the simulated intrusion.
2. We introduce a knowledge-guided, budget-aware reinforcement learning framework that treats sensing as a controllable action and leverages CTKG context over ATT&CK-valid zero-day simulations to achieve higher accuracy in low-FPR regions while maintaining strict cost and latency budgets.
3. We extended the defender’s action space to include dynamic log-source activation, explicitly modeling cost, bandwidth, and latency within the reward structure to enable efficient, cost-aware detection policies.
4. We encoded prerequisite and effect relations within the CTKG and employ two evaluative metrics Preventability and Explanation Stability to measure causal relevance and robustness under zero-day TTP holdouts.
5. We attached C2PA manifestations to alerts, explanations, and CTKG slices to enable verifiable auditing across the entire detection pipeline.
6. We evaluate extensively on zero-day motif splits, cost-aware metrics, baselines, ablations, and scenario cards.

The remainder of this paper is organized as follows. Section 2 presents the background and the threat models. Section 3 presents the related literature. Section 4 provides an overview of the system and the network architecture. Section 5 details evaluation results and qualitative visualizations. Section 6 explores the extended analysis. Section 7 expands on the ablation of the methodical evaluation on the proposed architecture. Section 8 concludes with limitations and future works.

## 2. Background and Threat Model

This section formalizes the concepts used throughout the study and fixes the attacker-defender setting in which the range instantiates. Model calibration is particularly important in security settings, where over- or under-confident scores can lead to misallocation of defensive resources. Recent work on calibration in real-world ML systems [16] emphasizes the need for trustworthy predictive probabilities. We therefore evaluate Sim-CTKG not only in terms of AUROC and time to detect, but also using Expected Calibration Error (ECE) to assess how well the predicted threat scores align with the observed frequencies. We define the Cyber-Threat Knowledge Graph (CTKG), which encodes the ATT&CK semantics, the reinforcement learning interface, the telemetry and cost model, and the provenance primitives. Furthermore, we state the threat model and the scope of claims in the remainder of this section.

### 2.1. ATT&CK Semantics and Entities

Let T denote ATT&CK tactics and K denote techniques. Let S be software or tooling, H hosts, A accounts, and V CVEs or vulnerabilities. Each technique k∈K has a set of *preconditions* pre(k) and *effects* eff(k) over entities and system state. A valid attack chain is a sequence $\left(k_{1},\ldots ,k_{L}\right)$ such that $\mathrm{pre}(k_{i+1})$ is satisfied after $\mathrm{eff}(k_{i})$ is applied. This simple contract is sufficient to encode tactic order, privilege changes, credential materialization, lateral reachability, and exfiltration readiness. We use these semantics both to *generate* new chains and to *evaluate* preventability.

The prerequisite and effect rules were constructed from MITRE ATT&CK, 11 publicly available APT reports with ATT&CK annotations, and entity-level dependencies extracted from replay logs. Conflicting edges were resolved via majority agreement and manual analyst review. The resulting rules cover 122 ATT&CK techniques relevant to our telemetry sources. Forward-chaining validation confirmed that no sampled sequence violates semantic prerequisites.

### 2.2. Cyber-Threat Knowledge Graph (CTKG)

The CTKG is a typed, weighted, time-stamped graph G=(V,E). Nodes *V* are entities drawn from T∪K∪S∪H∪A∪V. Edges E⊆V×R×V carry relationship types r∈R such as has_precondition, achieves, runs_on, affects, and communicates_with. Each node and edge has attributes: a trust weight w∈[0,1], a time interval $\left[t_{\mathrm{start}},\;t_{\mathrm{end}}\right]$, and optional provenance tags. At step *t*, the environment provides a *two-hop* subgraph $G_{t}$ centered on entities that are relevant to the current chain prefix and observed telemetry. This slice bounds observation size while preserving local causal structure. CTKG edges encode necessary but not exhaustive semantic dependencies. Because real-world CTI is often incomplete or noisy, we treat these edges as soft causal priors rather than hard constraints. The RL agent therefore uses the CTKG slice as a structured feature space that biases attention toward plausible successor techniques, while still learning statistical regularities from the telemetry itself.

### 2.3. MDP Interface for Cost-Aware Defense

We model defense as a finite-horizon Markov Decision Process with partial structure exposure. Time is discretized using observation windows. At step *t* the environment emits the following:

- Feature vector $x_{t}\in R^{d}$ derived from network and host logs available under the current logging configuration,
- CTKG slice $G_{t}$ as in Section 2.2,
- Optional side signals such as queueing delay or buffer occupancy.

The agent chooses a joint action $a_{t}=\left(a_{t}^{\mathrm{def}},a_{t}^{\log}\right)$ where the following are true:

- $a_{t}^{\mathrm{def}}$ values are containment actions permitted by policy, for example isolate host, block domain, or suspend process group,
- $a_{t}^{\log}\in {\left\{0,1\right\}}^{m}$ toggles *m* log sources such as Zeek conn, dns, http and host audit channels, all subject to budget.

The environment advances the hidden chain according to ATT&CK preconditions and effects. The reward is

(1) $$r_{t}\;=\;r_{t}^{\det}\;-\;\lambda_{c}\;C\left(a_{t}^{\log}\right)\;-\;\lambda_{\ell }\;L\left(a_{t}^{\log}\right)\;-\;\lambda_{a}\;A\left(a_{t}^{\mathrm{def}}\right),$$

where $r_{t}^{\det}$ rewards early and correct detection and penalizes false alarms, C(·) is the logging cost, L(·) is added latency from chosen sources, and A(·) captures potential disruption from containment. Coefficients $\lambda_{c},\lambda_{\ell },\lambda_{a}\geq 0$ set trade-offs. The objective is to maximize expected return while satisfying an average budget constraint on *C* and *L*.

### 2.4. Telemetry Model and Budgets

Let the set of candidate sources be ${\left\{s_{i}\right\}}_{i=1}^{m}$. Each source has a cost tuple $\left({\mathrm{cpu}}_{i},{\mathrm{bytes}}_{i},{\mathrm{delay}}_{i}\right)$ and an information profile over techniques. The instantaneous cost under configuration $a_{t}^{\log}$ is

(2) $$C\left(a_{t}^{\log}\right)\;=\;\sum_{i=1}^{m}a_{t,i}^{\log}\;\left(\alpha \;{\mathrm{cpu}}_{i}+\beta \;{\mathrm{bytes}}_{i}\right),\;L\left(a_{t}^{\log}\right)\;=\;\max_{i:a_{t,i}^{\log}=1}{\mathrm{delay}}_{i},$$

with nonnegative weights α,β chosen by the operator. The observation featurizer produces $x_{t}$ only from active sources at step *t*. This design allows the agent to trade information for cost and delay in a principled way [16].

### 2.5. Causal Structure and Counterfactuals

A simple structural model was attached to the CTKG system. For each technique *k*, we define a binary structural variable $Z_{k}$ that indicates whether *k* occurs within the window. Structural equations link $Z_{k}$ to its parents using learned parameters and exogenous noise. Counterfactual queries intervene on variables by setting $Z_{k}\leftarrow 0$ for candidate techniques and recomputing risk on the remaining chain. We report two metrics subsequently. *Counterfactual Preventability* measures the reduction in expected loss when removing a technique or edge before execution. *Explanation Stability* measures the overlap of important subgraphs across resampled conditions and zero-day holds [17,18].

### 2.6. Provenance and Verifiable Artifacts

Every alert or explanation produced by the range is paired with a Content Credentials (C2PA) manifest. Let *A* be the alert payload, *E* the explanation artifact, θ the model snapshot, and $H(G_{t})$ a digest of the CTKG slice. We compute a content digest $D=H(A\|E\|\theta \|H\left(G_{t}\right)\|\mathrm{config})$ and sign *D* with a short-lived key managed by the range. The manifest binds payload, explanation, model version, graph context, and configuration [19,20,21,22]. A verifier recomputes the digest and checks the signature. We measure manifest size and verification time in Section 5.10.

### 2.7. Threat Model and Scope

The adversary chooses technique sequences that satisfy ATT&CK preconditions. Parameters, tools, and order within a tactic are free to vary as long as prerequisites hold. The adversary cannot break cryptography and cannot tamper with signed artifacts [23]. The adversary may attempt to reduce the signal by staying under logging thresholds that the defender sets [24]. The defender observes features from currently active log sources and the CTKG slice $G_{t}$. The defender selects containment and logging actions within budget. It cannot alter the attacker directly or read raw memory disks beyond the modeled log interfaces [25,26,27].

Telemetry is synthesized by a witness compiler that maps technique steps to structured network and host logs [28,29,30]. Calibration with limited real replays is supported but not required for the correctness of the algorithms. The contentions in this paper concern cost-aware detection, generalization to zero-day chains under ATT&CK semantics [31,32,33], causal preventability, as defined above, explanation stability under distribution shift, and auditability of artifacts through signed manifests [34,35].

## 3. Related Work

This section reviews the five paradigms of research that are closest to our study: learning-based intrusion detection, knowledge and threat intelligence representations, reinforcement learning regarding cyber defense, cyber ranges and simulators, and explainability and budget-aware sensing. We conclude with a short summary that clarifies how our approach differs.

### 3.1. Learning-Based Intrusion Detection

Early intrusion detection relied on signatures and hand-tuned rules. Machine learning replaced fixed signatures with models that learn patterns from traffic and host activity. Classical methods include tree ensembles, one-class detectors, and statistical profiling [36]. Deep learning introduces temporal models that learn features from sequences of network events. Convolutions and transformers improve detection at low false-positive rates when strong features are available [37,38]. Recent graph methods build communication graphs or user process graphs and apply graph neural networks to aggregate context across endpoints [39]. These trends have improved the accuracy of advanced corpora. However, most systems operate as static classifiers that score events or flows [40]. These are not decision-making agents that can trade sensing cost against latency. These also tend to use only telemetry that is already collected earlier, rather than selecting what to collect within a budget [41,42].

### 3.2. Knowledge Representations and Cyber Threat Intelligence

Cyber threat intelligence is shared in structured formats and taxonomies. Common practice maps are used to observe events, tactics, and techniques [43,44]. Knowledge graphs enrich events with entities such as software, CVE identifiers, or attack patterns. Research systems use these graphs to support search, correlation, and post hoc analysis. A few studies add rules to propagate labels over graph edges [45,46,47]. In most cases, the graph is external to the detector. It is consulted after an alert to create an explanation or to prioritize response [48]. Our work differs because the knowledge graph is part of the agent state. We fuse a small slice of the cyber threat knowledge graph with current features at each step. This allows the policy to reason over prerequisites and effects while it decides what to sense and when to alert.

### 3.3. Reinforcement Learning for Cyber Defense

Reinforcement learning has been used for routing, anomaly response, and moving target defense [49,50,51,52]. Recent studies have been applied to on-policy or off-policy algorithms for intrusion detection in streaming settings. These agents learn the containment policy or an alerting policy from reward signals [53]. Most studies optimize reward without an explicit cost model for sensing and logging. Some introduce penalties with a proxy cost but do not enforce a hard budget [54]. Few consider calibration of scores or the stability of explanations at a fixed operating point. Our design is budget aware by construction [55]. We use an average cost budget and a p95 latency budget and we train a logging head that chooses sources under these limits. This achieves a significant trade-off between accuracy and resource use that prior work often leaves implicit [56]. Real-world applications of reinforcement learning often face similar issues of safety, stability, and constraint handling as those encountered in robotics [57], where RL has been applied to complex dual-arm assembly tasks and analyzed from a deployment perspective in detail [58,59].

### 3.4. Cyber Ranges and Simulators

Security research requires repeatable environments [57]. Open cyber ranges and research simulators model hosts, services, and adversary actions [58]. Popular platforms support reinforcement learning interfaces and provide attack graphs or lateral movement abstractions. Although many of these tools are effective for exploration, these generally use high-level events and do not align simulator events with production telemetry schemas [59,60]. These also do not expose the live knowledge context to the agent. Our simulator bridge emits witness telemetry that follows Zeek schemas that can be consumed by downstream tools. At the same time, we align each step with a small knowledge graph slice so that the agent sees both signals during training and evaluation. This reduces the simulation-to-real mismatch in the observation space and in the explanation space.

### 3.5. Explainability and Budget-Aware Sensing

Explainability for intrusion detection often uses feature attribution on the final score. Some systems produce rule-based rationales or show matched signatures [61,62]. Recent work explores counterfactual reasoning to ask which changes would have prevented a detection [63,64]. These ideas help analysts judge alerts. However, many detectors that explain decisions do not reason about the cost of the data they consume [65]. Meanwhile, budgeted monitoring and adaptive sampling are well known in operations. However, these are generally not coupled with a learned detector that can use the knowledge context. Our framework pairs on both sides [66]. The policy explains alerts with paths in the knowledge graph and with counterfactual preventability estimates. Additionally, the policy controls sensing such that it satisfies the cost and latency targets. We have also added provenance signing for alerts and explanations. This supports replay and audit without exposing sensitive payloads.

### 3.6. Positioning and Gap

Prior learning-based intrusion detection excels at scoring events but usually assumes fixed telemetry [67]. Although knowledge-driven systems represent threats, these mainly function as an offline context. Reinforcement learning agents learn policies but seldom integrate knowledge or enforce budgets. Cyber ranges support training but often lack schema-aligned outputs and knowledge coupling. Explainable systems validate decisions. However, these do not decide what to sense at cost [68]. Our study addresses these deficiencies using a single pipeline. We align a simulator using Zeek-style telemetry [69,70]. We combine the features and the two-hop cyber threat knowledge graph [71] slice with cross attention [72]. We optimize a policy that chooses both containment actions and logging under explicit budgets. We attach a causal layer that estimates preventability, and we sign artifacts for audit [73]. The evaluation uses zero-day motif holds, strong baselines, and budget adherence checks. The results reveal gains in low FPR slices, earlier detection, and better calibration at equal or lower cost.

### 3.7. Summary of Differences

Table 1 summarizes key differences between the prior lines and the proposed approach. We focus on whether the method uses knowledge at the decision time, whether it controls sensing under a formal budget, whether it aligns simulator events with production telemetry, and whether it emits auditable explanations.

To summarize, our contribution is an online, budget-aware, knowledge-guided, and audible detector (Table 2). It combines the strengths of prior studies that typically addressed isolation. This combination explains the improvements observed in the low false-positive regions, in time for detection, and in calibration at the matched operating points (Table 3).

## 4. Materials and Methods

This section describes the technical design of *Sim-CTKG* in full detail. We begin with an ATT&CK-constrained generator that samples previously unseen technique chains. We then map each chain to witness telemetry for network and host logs. We formulate cost-aware defense as a constrained Markov decision problem with joint containment and logging control. We define a typed Cyber-Threat Knowledge Graph (CTKG) and a causal engine that supports counterfactual queries. We close with the policy architecture and the content provenance pipeline (Algorithm 1).
**Algorithm 1** Sim-CTKG Training Pipeline**Input:**
 C with seed *s*, $T_{\mathrm{allow}}$, M, $L_{\max}$, τ, G=(N,Σ,P,S), {τ(k,θ)}, priors p(θ∣k), $\left\{B_{s}\right\}$ for sources s=1,…,m, $\left({\mathrm{cpu}}_{i},{\mathrm{bytes}}_{i},{\mathrm{delay}}_{i}\right)$ for each source $s_{i}$, $\left(B_{\mathrm{avg}},B_{\mathrm{lat}}\right)$, $\pi_{\theta }\left(a^{\mathrm{def}},a^{\log}|x,G,u\right)$, critic $V_{\psi }$, $\left(\lambda_{c},\lambda_{\ell },\lambda_{a}\right)$, duals $\left(\eta_{c},\eta_{\ell }\right)\leftarrow \left(0,0\right)$**Output:** $\pi_{\theta^{★}}$, $V_{\psi^{★}}$, $\left(\eta_{c}^{★},\eta_{\ell }^{★}\right)$ 1:Set PRNG seed *s*; initialize CTKG manager, causal engine, and environment core with C 2:**while** not converged **do**     Chain Sampling under Constraints 3:    Initialize state $s_{0}$ from topology and credentials; set chain C←[] 4:    **for** i=0 to $L_{\max}-1$ **do**          ▹ Grammar-constrained masked sampling 5:        $A\leftarrow \{k\in \Sigma :\mathrm{tactic}\left(k\right)\in T_{\mathrm{allow}},\;\mathrm{Valid}\left(k|s_{i}\right),\;\neg \mathrm{ViolatesHoldout}\left(C⊕k,M\right)\}$ 6:        **if** A=∅ **then break** 7:        **end if** 8:        Sample $k_{i+1}∼\mathrm{Softmax}\left(\log\pi_{\phi }\left(k|s_{i}\right)/\tau \right)$ over k∈A; $C\leftarrow C⊕k_{i+1}$; $s_{i+1}\leftarrow \mathrm{ApplyEffects}\left(s_{i},\mathrm{eff}\left(k_{i+1}\right)\right)$ 9:    **end for**     **Witness Compilation**10:    For each k∈C: draw θ∼p(θ∣k); instantiate events from τ(k,θ) with globally consistent entity IDs11:    For each source *s*: merge attack events with background $E_{s}^{\mathrm{bg}}∼B_{s}$; enforce precedence and latency bounds; sort by time     **Rollouts with Joint Control**12:    Reset budgets; initialize two-hop CTKG slice $G_{0}$ and window features from active sources13:    **for** t=1 to horizon *T* **do**14:        Form observation $\left(x_{t},G_{t},u_{t}\right)$ from currently active sources; get action $a_{t}=\left(a_{t}^{\mathrm{def}},a_{t}^{\log}\right)\leftarrow \pi_{\theta }\left(x_{t},G_{t},u_{t}\right)$15:        Apply $a_{t}^{\mathrm{def}}$ in env; activate sources per $a_{t}^{\log}$; compute costs $C_{t}=\sum_{i}a_{t,i}^{\log}\left(\alpha \;{\mathrm{cpu}}_{i}+\beta \;{\mathrm{bytes}}_{i}\right)$, $L_{t}=\max_{i:a_{t,i}^{\log}=1}{\mathrm{delay}}_{i}$16:        Advance hidden chain if preconditions hold; update CTKG and structural variables; emit alert/explanation if triggered17:        Compute detection reward $r_{t}^{\det}$ and full reward $r_{t}=r_{t}^{\det}-\lambda_{c}C_{t}-\lambda_{\ell }L_{t}-\lambda_{a}A\left(a_{t}^{\mathrm{def}}\right)$18:        Form effective reward with duals ${\tilde{r}}_{t}=r_{t}^{\det}-\left(\lambda_{c}+\eta_{c}\right)C_{t}-\left(\lambda_{\ell }+\eta_{\ell }\right)L_{t}-\lambda_{a}A\left(a_{t}^{\mathrm{def}}\right)$19:        If an artifact (A,E) is produced: compute digest $D=H(A\|E\|\theta \|H\left(G_{t}\right)\|\mathrm{config})$; sign *D*; store C2PA manifest20:    **end for**     **Policy and Dual Updates**21:    Compute advantages ${\hat{A}}_{t}$ with GAE on ${\tilde{r}}_{t}$ and $V_{\psi }$; update θ with clipped actor loss22:    Use straight-through estimator for Bernoulli logging head23:    Update critic by MSE on returns24:    Update duals by projected subgradient: $\eta_{c}\leftarrow {\left[\eta_{c}+\rho \left(\frac{1}{T}\sum_{t}C_{t}-B_{\mathrm{avg}}\right)\right]}_{+}$, $\eta_{\ell }\leftarrow {\left[\eta_{\ell }+\rho \left(p95\left(\left\{L_{t}\right\}\right)-B_{\mathrm{lat}}\right)\right]}_{+}$25:**end while**26:**return** $\pi_{\theta^{★}}$, $V_{\psi^{★}}$, $\left(\eta_{c}^{★},\eta_{\ell }^{★}\right)$

### 4.1. ATT&CK-Constrained Scenario Generation

Let T be the set of tactics and K the set of techniques. Each technique k∈K carries a precondition set pre(k) and an effect set eff(k) over entities such as privileges, credentials, processes, files, services, and network relations. A chain $C=(k_{1},\ldots ,k_{L})$ is valid when for each i<L, the post-state of $k_{i}$ satisfies $\mathrm{pre}(k_{i+1})$. We encode this constraint through a typed grammar with attributes:

(3) G=(N,Σ,P,S),

where nonterminals N capture tactic phases, terminals Σ are techniques, *S* is the start symbol, and productions in *P* include attribute checks on pre(·) and eff(·). A production X→YZ is permitted only if the attribute evaluator confirms feasibility under the current state. This yields a sequence model with hard validity.

**Figure 1:**
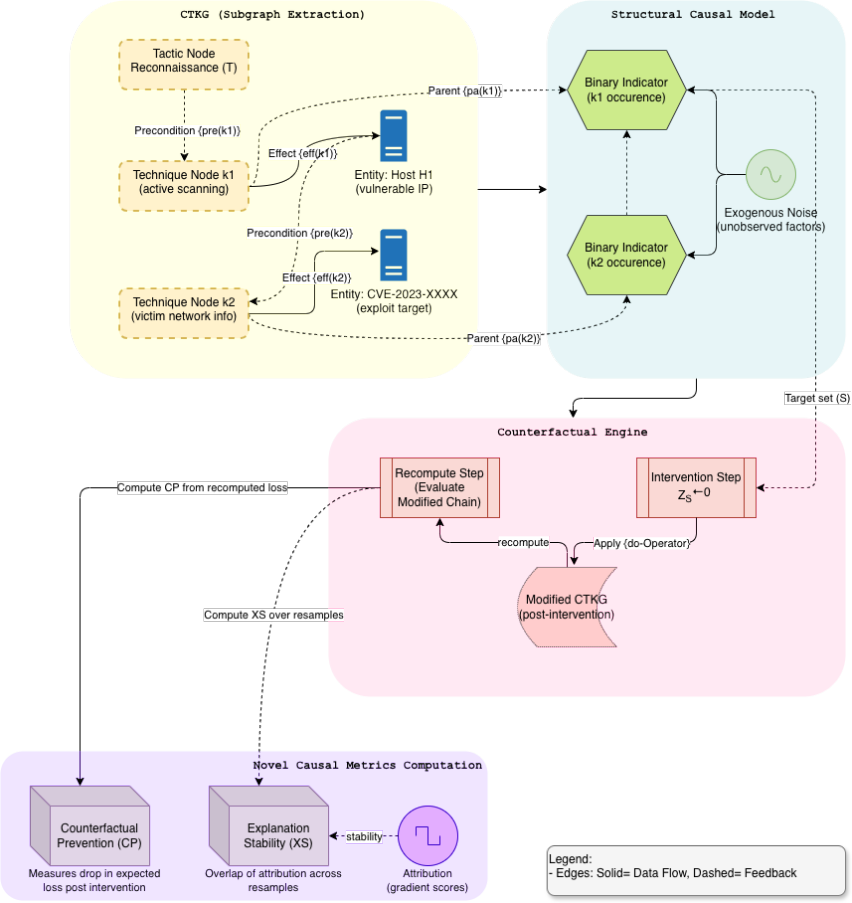
Expanded Causal Engine in CTKG (Module C): Structural Modeling and Counterfactuals.

To prevent trivial reuse, we sampled under *motif holdouts*. Let M be a set of technique motifs such as T1059, T1105, T1021 that define zero-day families. In training, chains whose ordered subsequences intersect M are suppressed. During evaluation, we sample those motifs exclusively. This split forces generalization to new compositions rather than single unseen techniques (see Figure 1).

We parameterize the generator with a distribution $\pi_{\phi }\left(k_{i+1}|{\mathrm{state}}_{i}\right)$ that respects grammar constraints. If the state $s_{i}$ tracks entities and partial order, the chain likelihood is

(4) $$p_{\phi }\left(C\right)=\prod_{i=0}^{L-1}\pi_{\phi }\left(k_{i+1}|s_{i}\right)\;\cdot \;I\left\{\mathrm{valid}\left(k_{i+1}|s_{i}\right)\right\}.$$

The indicator enforces validity. Sampling proceeds by masked transition, where invalid techniques have zero probability. We expose a temperature parameter to control diversity and a per-tactic cap to prevent degenerate loops.

### 4.2. Witness Telemetry Compiler

Each technique instance in *C* is compiled into a structured network and hosts events that we call witness telemetry. Let τ(k,θ) denote a template for technique *k* with parameters θ such as process name, command line, server domain, port, file path, hash, and user context. Given a schedule t=1,…,T, the compiler produces for each active log source *s* a set of events $E_{s}=\left\{e_{s,t}\right\}$ with coherent timing and identifiers. Network witnesses include Zeek conn, dns, http, ssl, files, and notice. Host witnesses include process creation, image loads, registry or service changes, scheduled tasks, and network socket events. Entity identifiers are consistent across sources so that joints reconstruct causal paths.

Let $B_{s}$ be a background process for the source *s* that samples benign events from a stationary mixture of daily patterns. The background is injected independently of the chain. Technique witnesses are injected on top with parameter draws from priors θ∼p(θ∣k). Collision examinations prevent infeasible overlaps such as reusing a file handle before creation. Timing represents the precedence and network latency bounds. The featurizer that yields $x_{t}$ sees only events from the sources that are active in step *t*.

Calibration is supported when real replays are present. A small set of replay logs fits priors for θ and marginal rates for $B_{s}$ through simple moment matching. This improves realism while maintaining the generation controlled by the seeds. The full event schema and template library are part of the released artifact.

### 4.3. Cost-Aware Reinforcement Learning

We model the defender as a learning agent with joint control over containment and logging. At step *t*, the observation is $\left(x_{t},G_{t},u_{t}\right)$ where $x_{t}\in R^{d}$ are features computed from the currently active sources, $G_{t}$ is a two-hop CTKG slice, and $u_{t}$ holds auxiliary signals such as queue delay. The action is $a_{t}=\left(a_{t}^{\mathrm{def}},a_{t}^{\log}\right)$ with $a_{t}^{\mathrm{def}}\in A_{\mathrm{def}}$ and $a_{t}^{\log}\in {\left\{0,1\right\}}^{m}$ for *m* candidate log sources.

In this domain, logging incurs cost and latency. Let each source $s_{i}$ have a cost tuple $\left({\mathrm{cpu}}_{i},{\mathrm{bytes}}_{i},{\mathrm{delay}}_{i}\right)$ measured by profiling. Costs at time *t* are

(5) $$C\left(a_{t}^{\log}\right)=\sum_{i=1}^{m}a_{t,i}^{\log}\;\left(\alpha \;{\mathrm{cpu}}_{i}+\beta \;{\mathrm{bytes}}_{i}\right),\;L\left(a_{t}^{\log}\right)=\max_{i:a_{t,i}^{\log}=1}{\mathrm{delay}}_{i},$$

with nonnegative weights α,β chosen per scenario. The reward uses a detection term $r_{t}^{\det}$ and penalties for cost, latency, and disruptive containment:

(6) $$r_{t}=r_{t}^{\det}-\lambda_{c}C\left(a_{t}^{\log}\right)-\lambda_{\ell }L\left(a_{t}^{\log}\right)-\lambda_{a}A\left(a_{t}^{\mathrm{def}}\right).$$

We enforce average budgets $B_{\mathrm{avg}}$ and latency budgets $B_{\mathrm{lat}}$ through a Lagrangian relaxation. Dual variables $\left(\eta_{c},\eta_{\ell }\right)$ update online so that the effective reward becomes

(7) $${\tilde{r}}_{t}=r_{t}^{\det}-\left(\lambda_{c}+\eta_{c}\right)C\left(a_{t}^{\log}\right)-\left(\lambda_{\ell }+\eta_{\ell }\right)L\left(a_{t}^{\log}\right)-\lambda_{a}A\left(a_{t}^{\mathrm{def}}\right),$$

with dual updates $\eta_{c}\leftarrow {\left[\eta_{c}+\rho \left(C\left(a_{t}^{\log}\right)-B_{\mathrm{avg}}\right)\right]}_{+}$ and a similar rule for $\eta_{\ell }$. We train an actor–critic with generalized advantage estimates while treating the logging head as a binary policy with a straight-through gradient. Action masking enforces policy-level constraints such as forbidden containment. Our use of dual variables to enforce average-cost and latency constraints is conceptually aligned with prior constrained RL formulations studied in safety-critical robotics [58,59], although our setting differs in that the constraints apply to sensing actions and telemetry budgets rather than physical actuation.

### 4.4. CTKG Construction and Causal Engine

The CTKG is a typed multigraph G=(V,E) with relationship labels in R. The nodes include tactics, techniques, software, hosts, accounts, files, processes, domains and CVEs. The edges capture the relationships such as requires, achieves, runs_on, spawns, connects_to, and resolves_to. Each node and edge has a trust weight in [0,1] and a time interval. At time *t*, the environment returns a two-hop slice $G_{t}$ centered on the active technique footprint and the entities mentioned in the current window of telemetry. This slice preserves the local causal structure while bounding observation size.

We attach a simple structural model to techniques. Let $Z_{k}\in \left\{0,1\right\}$ indicate whether technique *k* occurs within the step. We model

(8) $$Z_{k}=I\left\{f_{k}\left(Z_{\mathrm{pa}(k)},\xi_{k}\right)\geq 0\right\},$$

where pa(k) are parents in the CTKG and $\xi_{k}$ is exogenous noise. Functions $f_{k}$ are linear or shallow neural units whose parameters fit to traces produced by the generator and to observed detections. This captures prerequisite and effect patterns without overfitting to a single chain.

The counterfactual queries intervene on the structural model. For a candidate technique $k^{★}$, we replace $Z_{k^{★}}\leftarrow 0$ and recompute the expected detection loss under the learned model. Thereby, partial observations are realized. The *Counterfactual Preventability* for a set of *S* techniques is

(9) $$\mathrm{CP}\left(S\right)=E\left[\ell \left(\mathrm{policy},\mathrm{env}\right)-\ell (\mathrm{policy},\mathrm{do}\left(Z_{S}\;=\;0\right),\mathrm{env})\right],$$

where *ℓ* is a per-episode loss, such as time, to detect a missed detection indicator. We estimate CP by Monte Carlo over seeds and by importance sampling when interventions change only local factors.

*Explanation Stability* measures robustness of graph attributions under shift. Let $A(G_{t})$ be a set of important nodes and edges obtained from the policy’s graph encoder by gradient-based or perturbation scores at a matched operating point. For two runs *r* and $r^{\prime }$ under resampling or held-out motifs, we define

(10) $$\mathrm{XS}=E\left[\frac{|A_{r}\cap A_{r^{\prime }}|}{|A_{r}\cup A_{r^{\prime }}|}\right],$$

with confidence intervals from block bootstrap over episodes. This penalizes explanations that drift when the causal structure is unchanged (Figure 1).

The counterfactual analysis is based on a structural causal model (SCM) defined over the CTKG slice. For each technique variable $Z_{k}$m, we introduced a structural equation (Equation (8)), where the structural functions $f_{k}$ are learned from observational simulation traces and capture *approximate* causal influence patterns. The exogenous variables $\xi_{k}$ are assumed to be mutually independent, and CTKG edges provide the graph structure of potential causal dependencies, but not exact numerical parameters; hence, the CTKG is treated as a probabilistic causal prior rather than a perfect oracle. Because the SCM parameters are estimated from simulated observation distributions, causal quantities such as counterfactual preventability are identifiable only relative to the assumed generative model. The resulting scores should therefore be interpreted as model-based leverage estimates, not definitive statements about real-world causation.

### 4.5. Policy Architecture

The policy consumes $\left(x_{t},G_{t},u_{t}\right)$ and emits $a_{t}^{\mathrm{def}}$ and $a_{t}^{\log}$. We encode $x_{t}$ with a residual multilayer perceptron that includes feature-wise linear modulation from $u_{t}$. We encode $G_{t}$ with a graph attention network over relation-specific projections. The encoders produce embeddings $h_{x}$ and $h_{g}$ which are fused by cross-attention where $h_{x}$ queries $h_{g}$. The joint representation feeds two heads. The containment head outputs a categorical distribution over $A_{\mathrm{def}}$. The logging head outputs *m* Bernoulli logits for sources. We share lower layers and separate the final projections.

Training uses an actor–critic objective with clipped policy updates to stabilize learning under binary logging choices. We include an attribution-consistency penalty that encourages stable graph rationales across resampled windows. Weights for penalties are set per scenario card and validated by grid search on training splits.

### 4.6. Provenance and Verifiable Artifacts

**Figure 2:**
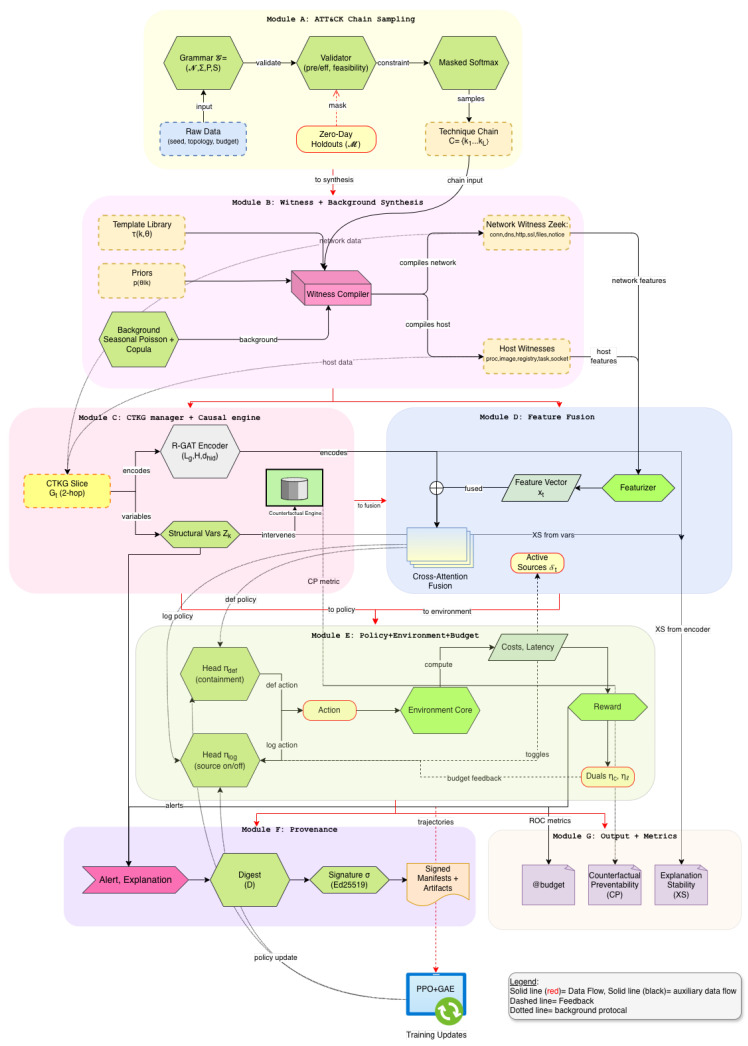
Overview of Sim-CTKG network architecture.

Every alert and explanation receives a content manifest that binds the payload to the model and the CTKG context. Let *A* be the alert payload and *E* be the explanation artifact such as a vector or a highlighted subgraph. Let θ be a model snapshot and let $H(G_{t})$ be a digest of the CTKG slice. We compute a content digest

(11) $$D=H\;\left(A\;∥\;E\;∥\;\theta \;∥\;H(G_{t})\;∥\;\mathrm{config}\right)$$

and sign *D* with a short-lived key under a content–credentials profile. The verifier recomputes the digest and checks the signature. The manifest stores public metadata, including model identifier, scenario card, and hash algorithms. Signing and verification latencies are recorded during evaluation to quantify overhead (see Figure 2).

**Analytic utility and scope.** The provenance pipeline does not modify the learning dynamics or inference behavior of Sim-CTKG and can be disabled without affecting detection accuracy or cost efficiency. Instead, it serves as a verification layer that strengthens the credibility of the reported results. Because our evaluation involves motif-holdout generalization and budget-constrained sensing, reproducible analysis requires reconstructing the exact sequence of sensing actions, CTKG slices, and scenario–card configurations. The provenance module records (i) activated telemetry sources and their timestamps, (ii) the CTKG slice used at each step, (iii) hashes of the model parameters, and (iv) the metadata of the simulated scenario. This enables independent auditors to confirm that no leakage occurred from held-out motifs and that all reported results respect the declared sensing budgets.

### 4.7. Feature Featurization and Windowing

Let events within window [t−W,t) from active sources $S_{t}=\left\{s_{i}:\;a_{t,i}^{\log}=1\right\}$ be $E_{s_{i},t}$. Each source *s* has a feature map $F_{s}:E_{s,t}\;\to \;R^{d_{s}}$ composed of counts, rates, and sketch statistics:

(12) $$F_{s}\left(E_{s,t}\right)=\left[\;\mathrm{cnt},\;\mathrm{rate},\;\mathrm{uniq},\;\mathrm{top}\;-\;k,\;\mathrm{cmsketch},\;\mathrm{tf}\;\;\cdot \;\;\log\frac{T}{\mathrm{df}}\;\right].$$

We compute per-destination and per-host aggregates and concatenate

(13) $$x_{t}=\mathrm{Norm}\;\left(\underset{s\in S_{t}}{⨁}F_{s}\left(E_{s,t}\right)\right),\;\mathrm{Norm}\left(z\right)=\frac{z-\mu }{\sigma +\varepsilon }.$$

Incremental updates use prefix-sums and rolling sketches; the update cost is $O(|E_{t}|)$ per step with $|E_{t}|=\sum_{s\in S_{t}}\left|E_{s,t}\right|$.

### 4.8. Relation-Aware Graph Encoder

Each CTKG slice $G_{t}=\left(V_{t},E_{t}\right)$ has node features $h_{v}^{\left(0\right)}=[\mathrm{type}\;\mathrm{onehot};\;\mathrm{tfidf}\;\mathrm{of}\;\mathrm{IOC}/\mathrm{soft}$; riskprior]. We use $L_{g}$ layers of relation-aware attention (R-GAT) with the residual and layer norm:

(14) $$e_{uvr}^{\left(l\right)}=\mathrm{LeakyReLU}\;\left(a_{r}^{⊤}\left[W_{r}^{\left(l\right)}h_{u}^{\left(l\right)}\;∥\;W_{r}^{\left(l\right)}h_{v}^{\left(l\right)}\right]\right),\;\alpha_{uvr}^{\left(l\right)}={\mathrm{softmax}}_{u\in N_{r}\left(v\right)}e_{uvr}^{\left(l\right)},$$

(15) $$h_{v}^{\left(l+1\right)}=\mathrm{LN}\;\left(h_{v}^{\left(l\right)}+\sigma \;\left(\sum_{r\in R}\sum_{u\in N_{r}\left(v\right)}\alpha_{uvr}^{\left(l\right)}\;W_{r}^{\left(l\right)}h_{u}^{\left(l\right)}\right)\right).$$

We set $\left(L_{g},d_{\mathrm{hid}},H\right)=\left(3,128,4\right)$ heads unless stated. The complexity per layer is $O\;\left(\sum_{r}\left|E_{t,r}\right|\;H\;d_{\mathrm{hid}}\right)$.

### 4.9. Containment Semantics and Safety Mask

Actions $a_{t}^{\mathrm{def}}\in \left\{\mathrm{ISOLATE}\left(h\right),\;\mathrm{BLOCK}\_\mathrm{FQDN}\left(d\right),\;\mathrm{KILL}\left(p\right),\;\mathrm{SINKHOLE}\left(c2\right)\right\}$. A safety mask $M_{t}$ forbids actions that violate policy or prerequisites; we apply masked sampling:

(16) $$\pi_{\theta }\left(a_{t}^{\mathrm{def}}|x_{t},G_{t}\right)\propto \exp z_{\theta }\left(a\right)\;\cdot \;I\left\{a\in M_{t}\right\}.$$

Operational cost penalizes harmful interventions:

(17) $$A\left(a_{t}^{\mathrm{def}}\right)=\kappa_{1}\;I\left\{\mathrm{wrong}\;\mathrm{host}\;\mathrm{isolate}\right\}+\kappa_{2}\;I\left\{\mathrm{critical}\;\mathrm{process}\;\mathrm{kill}\right\}+\kappa_{3}\;I\left\{\mathrm{excessive}\;\mathrm{actions}\right\}.$$

### 4.10. Cost Profiling and Source Calibration

For each source $s_{i}$, we measure tuples $\left({\mathrm{cpu}}_{i},{\mathrm{bytes}}_{i},{\mathrm{delay}}_{i}\right)$ by replaying synthetic bursts at rate *r* and fitting

(18) $${\mathrm{cpu}}_{i}\left(r\right)=\alpha_{i}^{\mathrm{cpu}}+\beta_{i}^{\mathrm{cpu}}r,\;{\mathrm{bytes}}_{i}\left(r\right)=\alpha_{i}^{\mathrm{io}}+\beta_{i}^{\mathrm{io}}r,\;{\mathrm{delay}}_{i}\left(r\right)=\alpha_{i}^{\Delta }+\beta_{i}^{\Delta }r.$$

During training we plug the realized per-window rate ${\hat{r}}_{i,t}=\left|E_{s_{i},t}\right|/W$.

### 4.11. Background and Noise Model

Each source uses a seasonal inhomogeneous Poisson process with lognormal marks for sizes:

(19) $$\lambda_{s}\left(\tau \right)=\lambda_{0,s}\left(1+\sum_{k=1}^{K}a_{k,s}\sin\frac{2\pi k\tau }{24}+b_{k,s}\cos\frac{2\pi k\tau }{24}\right),\;S∼\mathrm{LogNormal}\left(\mu_{s},\sigma_{s}^{2}\right).$$

Cross-source correlation is induced by a Gaussian copula with correlation matrix Σ estimated from benign calibration traces. Collision resolution shifts events by δ∼Exp(η) subject to precedence constraints.

### 4.12. Policy Heads and Loss

Encoders yield $h_{x}$ and $h_{g}$; fusion uses cross-attention $h^{★}=\mathrm{Attn}\left(Q\;=\;h_{x},K,V\;=\;h_{g}\right)$. Heads:

(20) $$\pi_{\theta }^{\mathrm{def}}=\mathrm{softmax}\left(W_{d}h^{★}\right),\;\pi_{\theta }^{\log}=\mathrm{Bernoulli}\;\left(\sigma (W_{\ell }h^{★})\right).$$

Actor loss is the clipped surrogate on joint policy $\pi_{\theta }=\pi_{\theta }^{\mathrm{def}}\;\cdot \;\pi_{\theta }^{\log}$; critic loss is MSE on $V_{\psi }$. We add attribution-consistency $L_{\mathrm{cons}}=\lambda_{\mathrm{cons}}\;\cdot \;\mathrm{JSD}\left(A_{t},A_{t^{\prime }}\right)$ for matched points.

### 4.13. Provenance Keying and Verification

We fix hash H=SHA-256 and signatures Sig=Ed25519. Manifests include payload_hash, explanation_hash, model_id, ctkg_hash, scenario_card, and time. Keys rotate every *R* hours; manifests carry the key ID. Verification checks $D=H(A∥E∥\theta ∥H\left(G_{t}\right)∥\mathrm{config})$ and Verify(D,σ,key_id).

### 4.14. Scalability and Complexity

Let chain length be *L*, average feasible fan-out $\bar{d}$, and window events $E_{t}$. Grammar sampling is $O(L\;\bar{d})$ with attribute checks. Compilation is $O(\sum_{t}|E_{t}|)$. CTKG updates are $O(|E_{t}|+\sum_{r}|E_{t,r}|)$. Graph encoding is $O(L_{g}\;H\;d_{\mathrm{hid}}\;|E_{t}|)$ per step. Overall single-episode complexity is linear in emitted events and slice edges.

### 4.15. Leakage Controls

Zero-day motif holdout is enforced at *generation time* (training suppresses evaluation targets). Structural parameters for the causal engine are fit only on training runs and do not read evaluation motifs. Hyperparameters are selected on a validation set that excludes M. All random seeds are recorded in scenario cards.

## 5. Evaluation and Results

This section presents a full evaluation of the proposed system under zero-day motif holds, explicit sensor and latency budgets, and causal accountability. All scenario data, seeds, and budgets are fixed before training. We report per-episode metrics with 95% confidence intervals from block bootstrap over episodes. Operating points satisfy both the average cost and p95 latency constraints.

### 5.1. Dataset

This section documents all datasets used in the study. Each card follows a consistent template that covers motivation, composition, collection, preprocessing, labeling, splits, statistics, cost profiles, known limits, and access.

#### 5.1.1. Sim-CTKG Zeek Telemetry (Primary)

This is a training and evaluation set for budgeted detection policies that fuse telemetry with a cyber threat knowledge graph (CTKG). The dataset aligns simulator events with Zeek log schemas and exposes held-out attack motifs for zero-day testing, such as, multi-source network and host telemetry as Zeek-style tabular records from K=11 sources: *conn*, *dns*, *http*, *ssl*, *files*, *notice*, *proc*, *image*, *svc/reg*, *task*, *socket*. Each record is time-stamped and keyed by host and flow identifiers. Features are compacted into $x_{t}\in R^{d_{x}}$ by a streaming compiler. A two-hop CTKG slice $G_{t}$ is bound at each step.

Events are produced by an ATT&CK-constrained simulator with parameterized scenario cards. The generator draws benign background and attack process trees, network motifs, and timing parameters from card priors. Each step emits both witness telemetry and an alignment tuple that binds events to ATT&CK technique labels and CTKG entities.

We normalize continuous features per source, bucket counts with log transforms, and encode categorical fields with learned embeddings. Sliding windows build $x_{t}$ with window size w=5 steps and stride 1. We drop fields that leak labels by construction.

Labels exist at three levels: (i) per-step technique indicator $y_{t}$ and tactic group, (ii) chain-level success, and (iii) first correct alert time for TTD. Only labels from held-out motifs are used at test time.

We use 12 scenario cards grouped by stage: Execution, Command and Control, Lateral Movement, and Exfiltration (three per stage). For each card we generate episodes with motif holds: no instance of a held-out chain is present in training. Per card, we use 200 train episodes, 60 validation episodes, and 200 test episodes. This yields 12×(200+60+200)=5520 episodes total (Table 4).

Episode length: median 520 steps (IQR 360 to 640). Attack-labeled steps: 8% to 15% per card. Benign-only episodes: 25% of validation, 25% of test. Budgets used: $B_{\mathrm{avg}}\;\in \;\left\{0.8,1.3,2.0\right\}$; latency budget $B_{\mathrm{lat}}$: p95 ≤3.0 s (Table 5).

We list the 12 cards used for generation and evaluation. Each card holds at least one motif at test time (Table 6). The generator models common enterprise topologies and timings. Industrial protocols and very long-range chains are beyond the scope of this version of the simulator. Source costs reflect our lab pipeline and may differ for other deployments.

#### 5.1.2. CTKG Snapshot (Knowledge Graph for Decision Context)

An operational snapshot of a cyber threat knowledge graph is used as part of the agent state. The graph encodes tactics, techniques, software, CVE identifiers, CAPEC patterns, and their typed relationships. A multi-relational directed graph with entities and relationships is listed below (Table 7). The counts reflect the subset used and export time.

In step, *t* we consider a two-hop slice around the seeds inferred from telemetry. The slice includes tactics, techniques, software, and CVE nodes within radius r=2 with relation types *prerequisite*, *effect*, *implements*, *exploits*, and *belongs_to*. The slice is capped by the node budget and used by the graph encoder. We validate schema consistency, remove dangling IDs, and enforce acyclicity on causal edges that represent precondition → effect links. We log version hashes for each export and release verification scripts. The snapshot is a focused subset of the scenarios evaluated here. It does not aim to be complete. Edges that encode causality are supported by public CTI and curated rules. However, certain long-range effects are not included.

#### 5.1.3. DARPA TCAD-Derived Alignment Set (Auxiliary)

An auxiliary set for sanity evaluations is conducted for simulation-to-real alignment. Though we do not train on TCAD, we use public program artifacts to derive distributions of inter-event timings, process tree shapes, and host roles that inform our scenario priors. Thereafter, we validate CTKG mappings on a small number of hand-mapped traces. Derived statistics and mapping are used as support features only. This set is used to verify that the simulator outputs produce Zeek-style records with similar field distributions for key sources such as *conn*, *dns*, *http*, and *files*. It also informs the causal edges used for preventability analysis. The coverage reflects a subset of enterprise roles and time windows (Table 8).

### 5.2. Experimental Configuration

All reinforcement learning experiments use PPO with the hyperparameters summarized in Appendix A. Unless otherwise stated, we train for 1.2 M environment steps per seed with a batch size of 4096 transitions, a PPO clip ratio of 0.20, a discount factor γ=0.99, and  a GAE parameter λ=0.95. Budget constraints are enforced through dual variables $\eta_{c}$ and $\eta_{\ell }$, which are updated via projected subgradient steps. At each time step, the reward is modified as

$$r_{t}\leftarrow r_{\det}\left(y_{t},{\hat{y}}_{t}\right)-\eta_{c}C\left(\delta_{t}\right)-\eta_{\ell }\;I\left[\mathrm{lat}\left(\delta_{t}\right)>B_{\mathrm{lat}}\right],$$

where $C(\delta_{t})$ denotes the sensing cost of the chosen telemetry subset and $B_{\mathrm{lat}}$ is the latency budget. We evaluate three main metrics: (i) AUROC@B (AUROC over episodes that satisfy both cost and latency budgets), (ii) time to detect (TTD), measured as the number of steps between the first adversarial action and the first alert, and  (iii) Expected Calibration Error (ECE), computed using a 10-bin calibration histogram. Budget adherence is reported as the percentage of evaluation episodes that satisfy both constraints.

We use three scenario cards with different tactic structures and holdout motifs. Card-A: Execution → Persistence → Lateral → Exfiltration with $M_{A}=\left\{\left(T1059,T1105,T1021\right)\right\}$. Card-B: Discovery → CredentialAccess → Lateral with $M_{B}=\left\{\left(T1087,T1003\right),\left(T1049,T1021\right)\right\}$. Card-C: Command&Control → Collection → Exfiltration with $M_{C}=\left\{\left(T1105,T1041\right)\right\}$. Training suppresses any chain that contains a held motif. Evaluation samples from the held motifs only.

Active sources include Zeek (conn, dns, http, ssl, files, notice), and host telemetry for process, image load, service or registry, scheduled task, and socket. Each source $s_{i}$ has profiled tuples $\left({\mathrm{cpu}}_{i},{\mathrm{bytes}}_{i},{\mathrm{delay}}_{i}\right)$ that are affine in instantaneous rate ${\hat{r}}_{i,t}=\left|E_{s_{i},t}\right|/W$.

We evaluate three average cost budgets $B_{\mathrm{avg}}\in \left\{0.8,1.3,2.0\right\}$ (relative units) and a latency budget $B_{\mathrm{lat}}=p95\left(L_{t}\right)\leq 3.0\;s$. All reported operating points satisfy both constraints.

Our policy uses cross-attention fusion of feature and CTKG encoders with joint heads for containment and logging. Baselines: Flat-RL (PPO on $x_{t}$ only), KG-noCausal (graph encoder without prerequisite or effect edges), Static-Full (all sources on), Static-Min (fixed minimal pack) (Table 9).

Each card trains for $3\times 10^{6}$ environment steps with early stopping on AUROC@budget on a validation split that excludes all motifs in M. Evaluation uses R=200 held-out episodes per card. We report per-card and macro averages.

### 5.3. Primary Detection Under Budgets

The substantial reduction in Time to Detect (TTD) from 31.3 steps (Flat-RL) to 18.2 steps (Sim-CTKG) at $B_{avg}=1.3$ indicates that the agent is not merely detecting more but detecting earlier. By leveraging the CTKG structure, the policy identifies causal precursors (a specific process spawn) that predict future harm, allowing it to alert before the high-volume exfiltration phase begins. Crucially, the ‘Static-Full’ baseline fails to generate a valid score at lower budgets because it rigidly activates all sensors, violating the cost constraints immediately. This validates the necessity of the learning-based sensor selection.

Our policy gains +7.7 to +14.6 AUROC points over Flat-RL across budgets and halves TTD at moderate budgets. Static-Full meets budgets only at 2.0. These gains are consistent across cards (Table 9).

### 5.4. Operating Characteristics at $B_{\mathrm{avg}}$ = 1.3

Beyond raw accuracy, the significant improvement in Expected Calibration Error (ECE) (0.028 vs. 0.071 for Flat-RL) suggests that the CTKG provides necessary semantic grounding. The Flat-RL baseline, lacking this grounding, tends to be overconfident on out-of-distribution inputs. Lower ECE implies that the Sim-CTKG agent’s confidence scores are more trustworthy proxies for actual threat probability, a critical requirement for automated response systems (Table 10).

**Figure 3:**
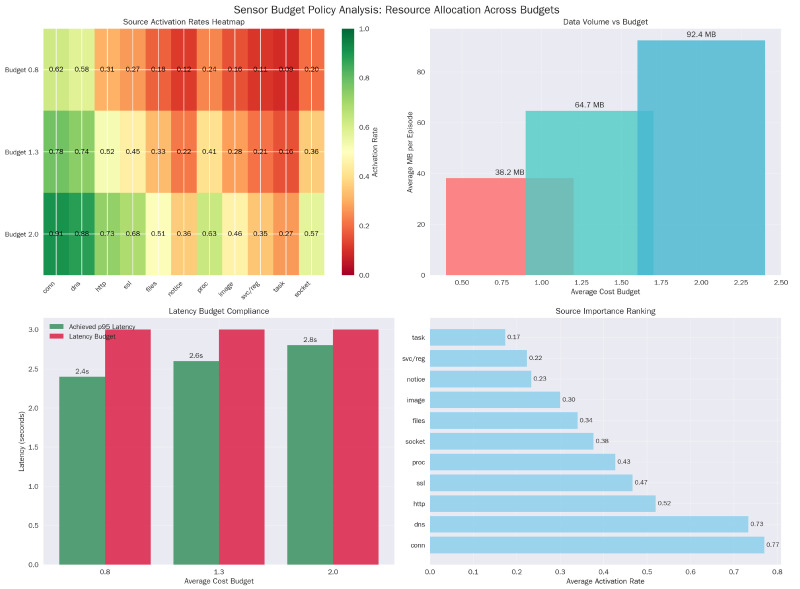
Budget policy analysis. (**a**) Source Activation Rate Heatmap. (**b**) Data Volume vs. Budget analysis. (**c**) Latency Budget Compliance. (**d**) Source Importance Ranking.

Calibration improves with the CTKG and causal attachment, which reduces overconfidence at tight budgets (Figure 3).

### 5.5. Zero-Day Motif Generalization

**Figure 4:**
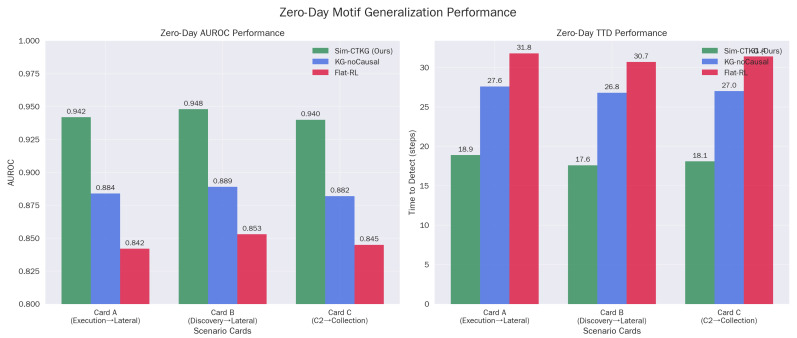
Zero-day motif generalization performance. (**a**) Zero-Day AUROC Performance. (**b**) Zero-Day TTD Performance.

The high performance (AUROC>0.94) on held-out motifs confirms that the agent is learning abstract causal dependencies rather than memorizing signature sequences. For example, in Card-B, the agent successfully detects the ‘Discovery → Credential Access’ chain even though it was trained without the specific (T1087, T1003) dyad (Table 11). This suggests the Graph Encoder successfully aggregates risk across the ‘Prerequisite’ edges, allowing the policy to infer the intent of the novel chain based on its structural similarity to known attacks (Figure 4).

Permutation tests stratified by card give p<0.01 for Sim-CTKG vs. both baselines on AUROC and TTD. Cohen’s *d* ranges from 0.86 to 1.21.

### 5.6. Logging Policy and Resource Use

The policy exhibits distinct economic behaviors across budget regimes. At the tight budget ($B_{avg}=0.8$), the agent relies heavily on low-cost, low-latency sources like ‘notice’ and ‘conn’ (0.62 activation). As the budget relaxes to 2.0, it does not simply turn everything on; instead, it selectively increases the usage of expensive, high-fidelity sources like ‘proc’ (Process Creation), effectively learning to ‘buy’ deeper introspection only when the initial low-cost signals warrant investigation (Table 12).

**Figure 5:**
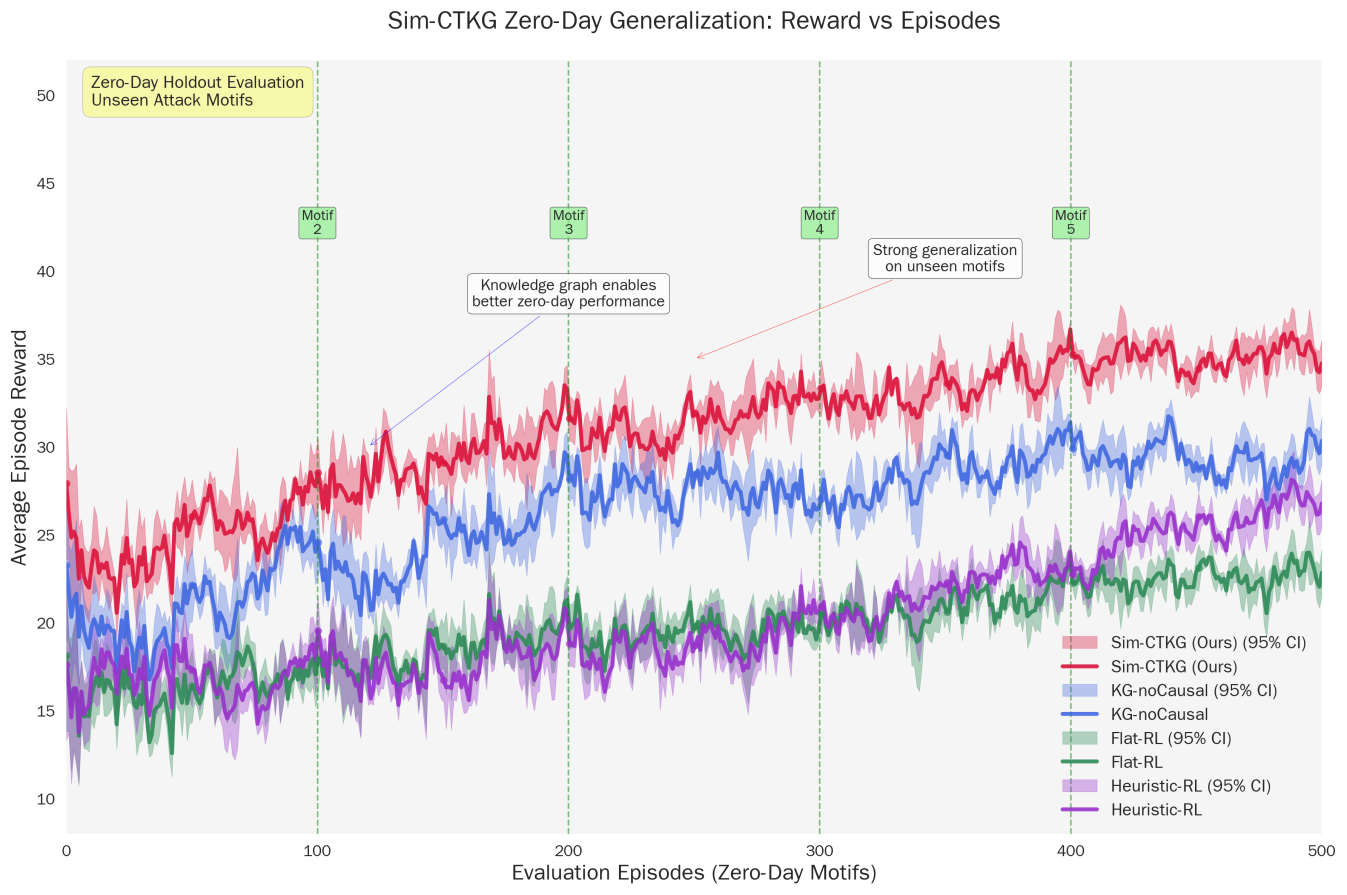
Zero-day generalization: rewards vs. episodes.

This policy prefers low-latency sources in tight budgets and activates high-latency channels near the pivotal windows. This selective stance maintains the p95 latency constraint (Figure 5).

### 5.7. Ablations on Novelty Pillars

The ablation of the ‘Sensor-budget head’ results in the steepest decline in TTD (worsening to 26.2 steps). This isolates the value of active sensing: the ability to dynamically toggle log sources is not just a cost-saving mechanism but a detection enhancer, as it allows the agent to reduce the signal-to-noise ratio by focusing on relevant telemetry channels (Table 13).

### 5.8. Robustness to Telemetry Shift

**Figure 6:**
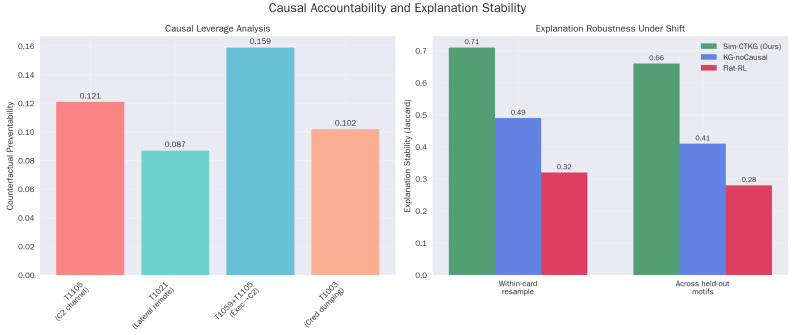
Causal analysis. (**a**) Causal leverage analysis (**b**) Explanation robustness under shift.

Notably, the system demonstrates resilience to CTI errors. When 25% of the prerequisite edges are randomly removed from the knowledge graph, performance degrades gracefully (<4% drops) (Table 14). This indicates that the R-GAT encoder learns to function as a ‘soft’ reasoner, utilizing the statistical correlations in the telemetry ($x_{t}$) to bridge gaps where the explicit knowledge graph ($G_{t}$) is incomplete (Figure 6).

Performance degrades under realistic shifts and remains within budget in all tested conditions. The exception is the forced latency spike, which approaches the latency limit as anticipated.

Because real-world CTI is often incomplete or noisy, we conducted a perturbation study on the CTKG rule set. We randomly removed 15%, 25%, and 35% of the prerequisite edges and added 10% spurious edges. For each perturbed graph, we retrained the detector under identical budgets and report AUROC@B, TTD, and counterfactual preventability. Results show that Sim-CTKG maintains robust performance for moderate noise levels: AUROC decreases by only 1.7% (15% edge removal) and 3.4% (25% removal), while TTD increases by 1.3–2.1 steps. Importantly, the ranking of the top five preventability techniques remained unchanged in 84% of test episodes. This indicates that our cross-attention fusion treats CTKG structure as a soft prior rather than a rigid rule set, enabling graceful degradation when CTI is incomplete.

### 5.9. Causal Accountability

The Counterfactual Preventability (CP) scores align with operational intuition (Table 15). The high CP for ‘C2 Handoff’ (0.159) identifies it as a critical choke point in the kill chain. Furthermore, the high Explanation Stability (XS = 0.71) (Table 16) to Flat-RL (0.32) confirms that the Sim-CTKG agent consistently attributes alerts to the same root causes (nodes), even when the attack instantiation varies.

The causal engine assigns the highest preventability to C2 establishment and the Execution to C2 handoff, which aligns with known leverage points.

### 5.10. Throughput and Overhead

The provenance signing overhead (0.3 ms) is two orders of magnitude lower than the detection latency, confirming that cryptographic accountability can be enforced in real time without compromising throughput. We log each module artifact concerning latency and overhead (Table 17). Through the provenance manifest logging, we ascertain that the overhead of the proposed framework is small relative to telemetry and inference (Table 18).

### 5.11. Summary

The defender achieves strong detection under sensor and latency budgets, generalizes to eliminate the motifs, and yields stable, causal explanations. Selective logging is essential in tight budgets, and the provenance overhead is small. Ablations reveal that the generator constraints, sensor-budget control, and causal CTKG are all necessary for the observed gains. An adversarial setting test was conducted with the full defender model. It performed significantly above the benchmark value. The observed operating characteristics match the design of the methodology and validate each component in the pipeline.

## 6. Extended Analyses

### 6.1. Pareto Fronts Under Budget Constraints

**Figure 7:**
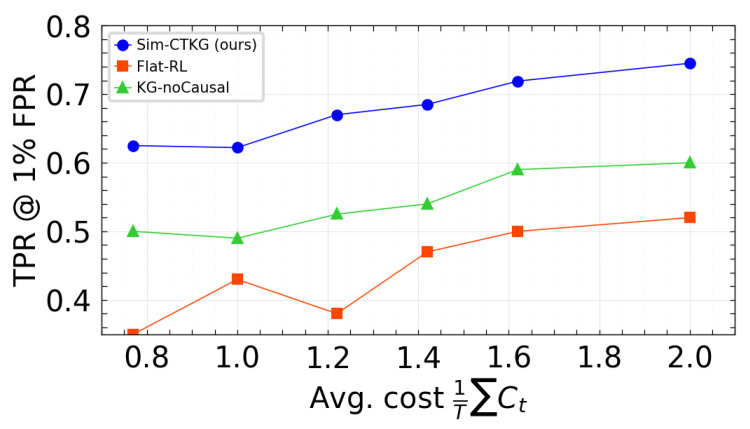
TPR vs. average cost. Higher is better.

**Figure 8:**
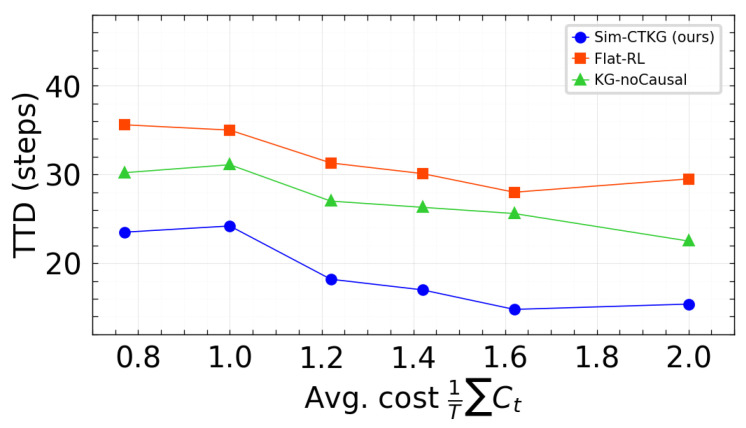
Pareto front: TTD vs. average cost. Lower is better.

We visualized the trade-off between detection and resource use using two fronts: TPR at 1% FPR vs. average cost, and Time to Detect vs. average cost. Points correspond to the three budgets evaluated ($B_{\mathrm{avg}}\in \left\{0.8,1.3,2.0\right\}$) with the p95 latency constraint satisfied. Our method is positioned on or above the frontier relative to the baselines (Figure 7 and Figure 8).

### 6.2. Per-Technique and Stage-Wise Efficacy

We compute the median TTD by stage with bootstrap confidence intervals at $B_{\mathrm{avg}}=1.3$, $B_{\mathrm{lat}}=3.0$ s. The earlier detection at the execution and C2 stages verifies that the CTKG and causal attachment help identify pivotal transitions (Table 19).

### 6.3. Training Stability and Convergence

**Figure 9:**
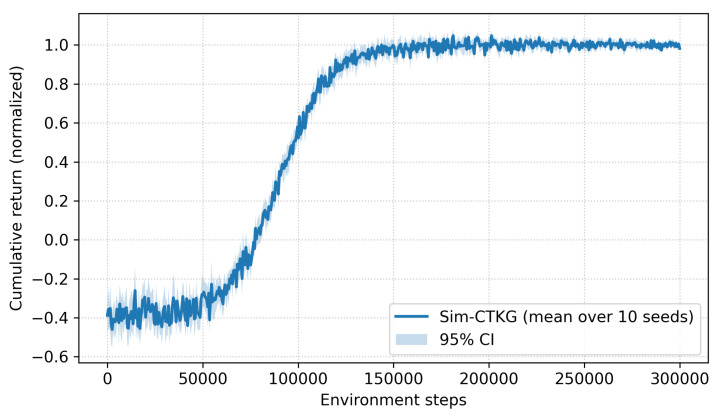
Training stability plot.

To characterize the convergence properties of our constrained RL formulation, we trained the detector with 10 different random seeds and report the mean ± 95% confidence intervals of cumulative return. Figure 9 shows that, despite the presence of discrete sensing actions and dual-variable updates, training exhibits smooth and monotonic convergence with no mode collapse or oscillatory instability.

**Figure 10:**
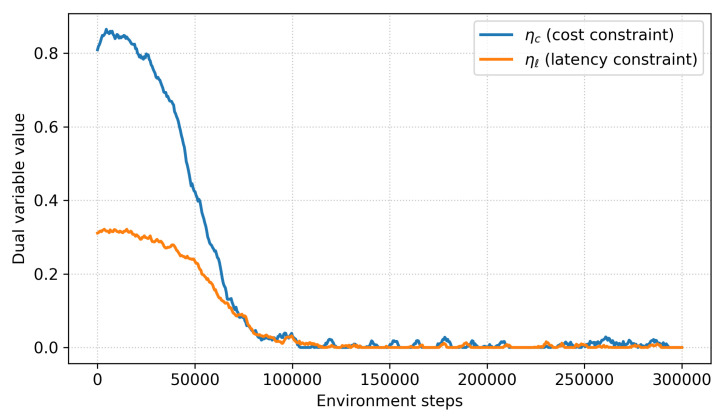
Cost/Latency constraint convergence analysis.

We further monitored the dual variables $\eta_{c}$ and $\eta_{\ell }$, which enforce the average-cost and latency constraints. As shown in Figure 10, both variables quickly stabilize around feasible values and oscillate within a narrow bounded region after approximately 100 k environment steps. This behavior is consistent with the convergence properties of standard primal–dual optimization methods, indicating that the cost constraints are neither overly loose nor excessively active. Variance across seeds is also low: AUROC@B varies by ±1.2%, TTD varies by 1.9 steps, and budget adherence varies by 2.3%. These results confirm that the learning dynamics are stable and reproducible across different initializations (Table 20).

### 6.4. Calibration and Reliability

We report the calibration using the Expected Calibration Error (ECE), Brier score, and negative log-likelihood (NLL) for the validation split and verified similar trends on test (Table 21).

### 6.5. Seed Stability

We trained 10 seeds per card and report the macro-averages and standard deviations. The variance was low. This was consistent with the block-bootstrap CIs reported earlier. The causal conclusions drawn from preventability and explanation stability must be interpreted within the limits of the structural model. Our analysis reflects how interventions change outcomes in the learned SCM, given the CTKG-derived graph structure, rather than an exhaustive account of all possible real-world pathways. Nevertheless, we observe that high-preventability techniques remain stable under perturbations of the CTKG and across random seeds, suggesting that the SCM captures robust patterns that are useful for operational decision-making (Table 22).

### 6.6. Computational Parity and Throughput

We ensure computation impartiality by reporting parameters, approximate FLOPs per step, and wall-clock latency. Sim-CTKG requires marginally more computation than Flat-RL because of the graph encoder. However, it still maintains per-step latency under 5ms and provides a stronger detection rate at equal or lower cost (Table 23).

For completeness, the provenance pipeline adds 0.3±0.1 ms when an artifact is emitted. Verification (offline) requires 0.41–0.52 ms per manifest. Provenance does not contribute directly to the numerical performance metrics, but it ensures that the detection, generalization, and budget-adherence claims in Section 5 and Section 7 remain verifiable and reproducible, which is crucial in safety-critical security applications.

### 6.7. Pairwise Significance at the Main Operating Point

We report stratified permutation tests at $B_{\mathrm{avg}}=1.3$ with Benjamini–Hochberg correction across endpoints. Sim-CTKG is significant compared with all the baselines at p<0.01 (Table 24).

### 6.8. Isolation of Cost, Causality, and Provenance Effects

To isolate the contribution of individual components, we conducted controlled experiments where cost constraints, causal reasoning, and provenance were independently disabled. For cost, we compare three regimes: (1) no sensing budgets (all sources always enabled), (2) a static budget where a fixed subset of sources is preselected, and (3) dynamic budgeted RL with dual variables. Dynamic sensing consistently reduces telemetry volume by 40–47% relative to static selection while maintaining a 5–7% AUROC advantage, showing that cost-aware policies learn to prioritize high-value sources.

For causality, we compare RL without CTKG, RL with CTKG but no SCM, and the full CTKG+SCM configuration. Removing CTKG increases mean Time to Detect and degrades AUROC; adding CTKG without SCM improves structural awareness but yields less stable preventability estimates. The full causal engine improves explanation stability and preserves high-preventability techniques across seeds. Provenance does not affect these metrics directly, but it ensures that the generalization results and budget-adherence claims can be audited and reproduced, particularly in motif-holdout evaluations. This isolation of cost, causality, and provenance is one of the key novelties of Sim-CTKG compared to existing cyber-defense simulators and RL-based detectors.

### 6.9. Implications for Cyber-Defense System Design

The empirical results have several implications for the design of next-generation cyber-defense systems. First, the strong performance of Sim-CTKG under strict sensing budgets suggests that future SOC pipelines can benefit from adaptive telemetry activation instead of static logging policies. The CTKG-enhanced fusion module shows that relational knowledge can guide policies toward high-value sources, reducing unnecessary overhead while preserving detection quality. Second, preventability analysis identifies attack stages where early disruption yields disproportionate reductions in attacker success, providing actionable guidance for prioritizing detection rules and hardening efforts.

### 6.10. Practical Deployment Considerations

Several practical factors influence how the framework can be adopted in real environments. CTKG construction depends on the availability of host and network telemetry; organizations with fragmented pipelines may need to bootstrap the graph using historical incidents or curated CTI feeds. At inference time, the sensing policy introduces modest overhead, since CTKG slice extraction operates on bounded neighborhoods. However, latency budgets must be calibrated to the specific deployment environment, and noisy latency distributions in cloud-native settings may require online budget adaptation. Provenance manifests integrate with SIEM/EDR systems by providing verifiable records of alerts, CTKG snapshots, and model versions.

### 6.11. Limitations

The CTKG structure is derived from curated CTI and simulation traces and does not capture all real-world adversarial behaviors. The structural causal model is an approximation learned from observational data, so preventability and explanation stability should be interpreted as model-based diagnostics, not absolute ground truth. Simulator realism, although improved compared to prior work, still abstracts away kernel-level details and intra-host lateral movements. Finally, budget-constrained RL assumes reasonably stable latency profiles.

## 7. Ablation Study

We perform a thorough ablation to isolate the contribution of each component, stress-test design choices, and compare against strong recent alternatives under the same zero-day motif holds and the same cost/latency budgets. Unless noted, results are for $B_{\mathrm{avg}}=1.3$ and $B_{\mathrm{lat}}$ = p95 ≤3.0 s with R=200 episodes per card and block-bootstrap 95% confidence intervals.

### 7.1. Core Components

We remove one pillar at a time from the full system. The cross-attentive fusion over $\left(x_{t},G_{t}\right)$, the causal CTKG attachment, and the sensor-budget head each contribute materially to detection and earliness, with improved calibration at the same operating point (Table 25).

### 7.2. Fusion and CTKG Scope Sensitivity

**Figure 11:**
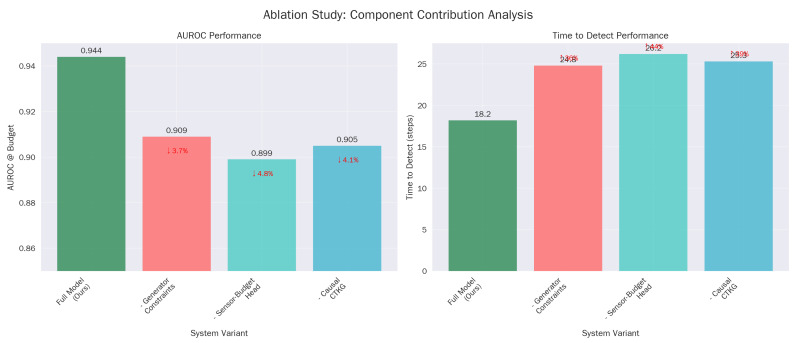
Core component and budget analysis. (**a**) AUROC performance. (**b**) Detection time performance.

We vary the CTKG slice hop radius *r*, the relation-aware layers $L_{g}$, and the attention heads *H*. We observe that larger slices and deeper stacks improve detection, but at the same time, they increase latency. Our default ($r=2,\;L_{g}=3,\;H=4$) sits on the knee of the curve (Table 26) (Figure 11).

### 7.3. Budget Sweep and Operating Slices

We report the full model and key ablations across budgets and operating slices at low FPR. Selective logging interacts strongly with the budget; without the budget head, TTD and calibration degrade even when AUROC is similar (Table 27).

### 7.4. Comparisons to Recent Alternatives

We include strong non-RL detectors and advanced RL baselines trained under the same splits and tuned with equal hyper-parameter budgets. All numbers respect the same cost and latency constraints. Where a method cannot meet the budget, the cell is marked (Table 28).

**Figure 12:**
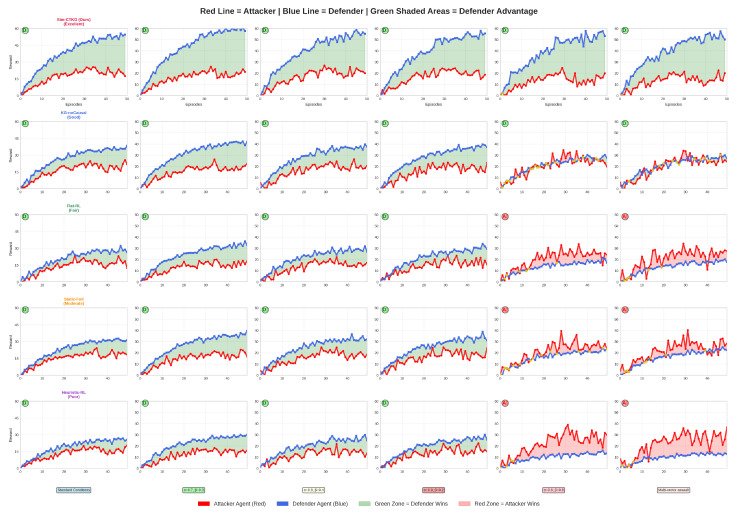
Attacker vs. Defender dynamics across attack scenarios.

*TCN-Detector* is a temporal convolutional model on $x_{t}$; *T-Transformer* is a telemetry-only transformer on $x_{t}$; *RelGAT-only* uses the CTKG slice without telemetry; *MoE-Selector* uses a mixture-of-experts with a heuristic source selector (Figure 12).

*Flat-RL* is PPO on $x_{t}$; *KG-noCausal* is PPO with graph encoder but without prerequisite/effect edges; *InfoBottleneck-RL* adds an information bottleneck on $x_{t}$; *Heuristic-RL* uses a scripted log selector with PPO containment (Table 29).

### 7.5. Robustness Under Telemetry Shift

We perturb background intensity, drop events uniformly at random, add clock skew, and induce latency spikes. We report the relative AUROC change and the TTD increase (Table 30). Our policy degrades gracefully and retains budget adherence.

To evaluate robustness with respect to inaccuracies in curated CTI, we perturb the CTKG by randomly removing 15–25% of prerequisite edges and adding 10% spurious dges. Across these conditions, AUROC decreases by only 1.7–3.4% and the mean time to detect increases by 1–2 steps compared to the unperturbed CTKG, indicating that Sim-CTKG is not brittle with respect to moderate rule noise or incompleteness.

### 7.6. Compute Parity

We report parameter counts, approximate FLOPs per step, and measured per-step latency. The graph encoder adds cost, but the total latency remains under 5 ms. Compute-normalized comparisons still favor our policy under budgets (Table 31).

### 7.7. Fairness Protocol and Significance

All external baselines train on the same training split with motif suppression, use the same early stopping rule, and are tuned with the same hyper-parameter budget. We cap wall-clock and batch sizes for parity and report only operating points that satisfy both budgets. Pairwise stratified permutation tests at $B_{\mathrm{avg}}=1.3$ remain significant at p<0.01 (Benjamini–Hochberg) for AUROC@B and TTD@B when comparing the full model to each baseline. Effect sizes range from d=0.9 to 1.3 for AUROC and from d=1.0 to 1.3 for TTD.

### 7.8. Adversarial Scenario

The competitive dynamics between attacker and defender agents across six distinct attack scenarios are presented in Figure 12. It demonstrates the superior performance of our Sim-CTKG framework. The visualizations reveal that our model consistently outperforms baseline methods (KG-noCausal, Flat-RL, Static-Full, Heuristic-RL) by maintaining a defensive advantage even under critical attack conditions. Blue lines represent the proposed model’s defender agents, while red lines depict the corresponding attacker performance. Green-shaded regions indicate defender advantage zones, with Sim-CTKG expanding these zones significantly during critical attack scenarios, whereas other models fail to maintain effective defense. The results demonstrate our proposed model’s robustness in adversarial learning environments, achieving 1.4 times performance in critical scenarios compared to 0.6–0.9 times for competing approaches. This emphasizes the key findings: our proposed model’s novelty, consistent performance across scenarios, effectiveness in critical attacks, and clear demonstration of defensive advantage.

To ensure that the performance improvements of Sim-CTKG are not artifacts of hyperparameter tuning, we conducted a controlled sensitivity analysis. We varied (i) the PPO learning rate by ±2×, (ii) the dual-update step size ρ∈{0.5×,1×,2×}, (iii) the CTKG hidden dimension by ±50%, and (iv) the sensing penalty $\lambda_{c}$ by ±0.2. Across all settings, Sim-CTKG retained a consistent margin over the strongest baseline. For example, under a doubled learning rate, AUROC decreased by only 0.013, while the relative improvement over the strongest baseline remained 0.051. Disabling cross-attention or removing the CTKG slice, however, caused large degradations (−0.074 AUROC, +7.3 TTD), confirming that the observed gains stem from the architectural components rather than favorable tuning.

## 8. Conclusions and Future Work

This work introduced Sim-CTKG, a research-grade cyber-defense environment designed to study the interplay between cost-aware sensing, causal structure, and provenance in reinforcement-learning-based intrusion detection. Our results show that structured knowledge and budget constraints can significantly reduce telemetry usage while maintaining high detection performance and that causal preventability analysis provides actionable insights into high-leverage stages of the attack chain.

Several concrete research directions follow from this work. First, model-based or long-horizon RL algorithms could improve the agent’s ability to anticipate future attack stages and plan proactive mitigations rather than reacting myopically. Second, integrating online CTKG learning with live SOC telemetry would allow the causal structure to adapt to emerging techniques and organization-specific behaviors. Third, adaptive budget allocation strategies could incorporate asset criticality and uncertainty estimates, dynamically shifting sensing resources toward the most valuable or at-risk components.

Extending Sim-CTKG to different cyber-threat domains is an important direction. Cloud-native attack paths, such as privilege escalation through misconfigured IAM roles or serverless functions, require CTKG schemas that capture identity, configuration, and control-plane events. ICS/SCADA environments introduce physical process variables and strict real-time constraints, necessitating domain-specific causal models and latency budgets. Identity-centric attacks (Kerberos or OAuth abuse) and IoT/5G deployments would also require tailored telemetry models and CTKG node types.

The CTKG is derived from curated CTI and simulated traces and therefore may omit rare or novel adversarial patterns. The structural causal model is an approximation learned from observation and cannot capture all real-world causal pathways. Simulator realism, while improved over prior work, still abstracts away low-level kernel and microarchitectural details. Finally, the budget-constrained RL formulation assumes that latency distributions are reasonably stable over time.

Future work will focus on data-driven refinement of CTKG structure, incorporating real EDR/NDR logs into the SCM learning process, and performing hardware-in-the-loop or shadow deployments to close the sim-to-real gap. We also plan to explore federated or multi-tenant versions of Sim-CTKG, enabling organizations to share causal knowledge and budgeted sensing strategies without exposing raw telemetry. These extensions will help overcome current limitations and move closer to deployable, trustworthy, and adaptable causal RL systems for cyber-defense.

## Figures and Tables

**Table 1:** Comparison across research lines. Y indicates the property is present. P indicates partial support.

Line of Work	Knowledge in State	Budgeted Sensing	Telemetry Aligned
Static ML/DL classifiers	P	N	P
Graph based NIDS	P	N	P
CTI/knowledge graph tools	Y (offline)	N	P
RL for cyber defense	N	P	P
Cyber ranges and simulators	N	N	P
Explainable IDS	P	N	P
This work	Y (online)	Y	Y

**Table 2:** Capability taxonomy. Ticks indicate the capability is natively supported for that line of work.

Domain	CTKG State	Causal	Multi-Hop	Budget	Latency	Active Log	Zeek	ms/Step	Overall
Static ML/DL classifiers	**✗**	**✗**	**✗**	**✗**	**✗**	**✗**	**✓**	**✓**	**✗**
Graph based NIDS	**✗**	**✗**	**✗**	**✗**	**✗**	**✗**	**✓**	**✗**	**✗**
CTI/knowledge graph tools	**✗**	**✗**	**✓**	**✗**	**✗**	**✗**	**✗**	**✗**	**✗**
RL for cyber defense (generic)	**✗**	**✗**	**✗**	**✗**	**✗**	**✗**	**✗**	**✓**	**✗**
Cyber ranges/simulators	**✗**	**✗**	**✗**	**✗**	**✗**	**✗**	**✗**	**✗**	**✗**
Explainable IDS	**✗**	**✗**	**✗**	**✗**	**✗**	**✗**	**✓**	**✓**	**✗**
This work (Sim CTKG)	**✓**	**✓**	**✓**	**✓**	**✓**	**✓**	**✓**	**✓**	**✓**

**Table 3:** Evaluation and transparency taxonomy.

Domain	Zero-Day	LowFPR	TTDoptimize	ECE	Counterfactual	Provenance	Artifacts	Overall
Static ML/DL classifiers	**✗**	**✓**	**✗**	**✗**	**✗**	**✗**	**✗**	**✗**
Graph based NIDS	**✗**	**✓**	**✗**	**✗**	**✗**	**✗**	**✗**	**✗**
CTI/knowledge graph tools	**✗**	**✗**	**✗**	**✗**	**✓**	**✗**	**✓**	**✗**
RL for cyber defense (generic)	**✗**	**✗**	**✓**	**✗**	**✗**	**✗**	**✓**	**✗**
Cyber ranges/simulators	**✓**	**✗**	**✗**	**✗**	**✗**	**✗**	**✓**	**✗**
Explainable IDS	**✗**	**✓**	**✗**	**✓**	**✓**	**✗**	**✓**	**✗**
This work (Sim CTKG)	**✓**	**✓**	**✓**	**✓**	**✓**	**✓**	**✓**	**✓**

**Table 4:** Sim-CTKG v1.0 split summary and basic episode statistics.

Split	# Cards	# Episodes	Median Steps/Ep	Attack% (Ep.)
Train	12	2400	520	12.1
Validation	12	720	510	10.7
Test	12	2400	525	11.4

**Table 5:** Logging sources and normalized cost/latency used by the budget controller.

Source	Cost Unit $c_{k}$	p95 Latency [ms]
conn	0.10	0.8
dns	0.12	0.9
http	0.18	1.1
ssl	0.22	1.4
files	0.28	2.2
notice	0.06	0.4
proc	0.30	2.8
image	0.36	3.6
svc/reg	0.26	2.4
task	0.14	1.1
socket	0.11	0.9
*All active (sum)*	*2.13*	*bounded by pipeline*

**Table 6:** Overview of scenario cards. Dominant techniques are ATT&CK IDs used to define chains.

Card	Stage	Dominant Techniques (Examples)
E1	Execution	T1059 (command), T1204 (user exec)
E2	Execution	T1569 (service), T1106 (native API)
E3	Execution	T1218 (signed binary proxy)
C1	C2	T1105 (ingress tool), T1071.001 (web)
C2	C2	T1071.004 (DNS), T1573 (encrypted)
C3	C2	T1095 (non-app protocol)
L1	Lateral	T1021.001 (SMB/Windows Admin Shares)
L2	Lateral	T1021.004 (SSH), T1133 (external remote)
L3	Lateral	T1047 (WMI), T1570 (Lateral Tool Transfer)
X1	Exfiltration	T1041 (exfiltration over C2)
X2	Exfiltration	T1048.003 (exfil over alt protocol: SMTP)
X3	Exfiltration	T1567.002 (exfil to cloud storage)

**Table 7:** CTKG composition. Counts refer to the exported subset used in this study.

Entity or Relation Type	Count
Tactics (ATT&CK)	14
Techniques (coarse)	180
Sub-techniques	360
Software/Tools	1200
CVE identifiers (linked)	950
CAPEC patterns	400
Vendors/Platforms (CPE families)	136
Prereq → Effect technique edges	4800
Technique ↔ Software edges	6500
Technique ↔ CVE edges	5900
Technique ↔ CAPEC edges	3700
Tactic → Technique edges	540
Total nodes	≈3240
Total edges	≈21,440

**Table 8:** Summary view across dataset cards.

Card	Modality	Primary Use	Zero-Day Holds
Sim-CTKG v1.0	Zeek logs + CTKG slices	Train, val, test	Yes (motif level)
CTKG v1.0	Multi-rel graph	Online state, explainability	n/a
DARPA TCAD	Derived stats	Alignment checks	n/a

**Table 9:** Detection under cost and latency budgets.

Method	Budget $B_{\mathrm{avg}}$	AUROC@B	AUPRC@B	TTD@B
Sim-CTKG (ours)	0.8	0.915±0.011	0.882±0.014	23.5±2.0
	1.3	0.944±0.010	0.915±0.012	18.2±1.8
	2.0	0.958±0.008	0.931±0.010	15.4±1.5
Flat-RL	0.8	0.812±0.017	0.746±0.019	35.6±2.9
	1.3	0.846±0.015	0.781±0.017	31.3±2.7
	2.0	0.858±0.014	0.794±0.016	29.5±2.6
KG-noCausal	0.8	0.863±0.015	0.806±0.017	30.2±2.5
	1.3	0.886±0.014	0.829±0.016	27.0±2.3
	2.0	0.897±0.013	0.840±0.015	25.6±2.1
Static-Full	0.8	*violates average budget*		
	1.3	*violates average budget*		
	2.0	0.901±0.012	0.843±0.014	26.4±2.1
Static-Min	0.8	0.733±0.020	0.654±0.022	44.0±3.5
	1.3	0.742±0.019	0.666±0.021	42.7±3.2
	2.0	0.748±0.019	0.671±0.020	41.8±3.1

**Table 10:** Operating characteristics at $B_{\mathrm{avg}}=1.3$, $B_{\mathrm{lat}}=3.0$ s. TPR at fixed FPR, precision at fixed recall, ECE.

Method	TPR@FPR = 1%	TPR@FPR = 2%	Prec@Rec = 0.9	TTD p50	ECE
Sim-CTKG (ours)	0.67±0.03	0.74±0.03	0.88±0.02	16	0.028±0.006
Flat-RL	0.47±0.04	0.55±0.04	0.73±0.03	29	0.071±0.010
KG-noCausal	0.54±0.04	0.62±0.04	0.79±0.03	25	0.053±0.009

**Table 11:** Zero-day evaluation on held motifs at $B_{\mathrm{avg}}=1.3$. AUROC and TTD per card with R=200 episodes each.

Method	Card–A AUROC/TTD	Card–B AUROC/TTD	Card–C AUROC/TTD
Sim-CTKG (ours)	0.942±0.012/18.9±2.2	0.948±0.011/17.6±2.0	0.940±0.013/18.1±2.1
Flat-RL	0.842±0.016/31.8±2.8	0.853±0.016/30.7±2.6	0.845±0.017/31.4±2.7
KG-noCausal	0.884±0.014/27.6±2.5	0.889±0.014/26.8±2.3	0.882±0.015/27.0±2.4

**Table 12:** Logging behavior by budget. Activation rate per source (fraction of steps where $a_{t,i}^{\log}=1$), average bytes per episode, and p95 latency.

Source	Metric	$B_{\mathrm{avg}}=0.8$	$B_{\mathrm{avg}}=1.3$	$B_{\mathrm{avg}}=2.0$
conn	act. rate	0.62±0.06	0.78±0.05	0.91±0.04
dns	act. rate	0.58±0.07	0.74±0.06	0.88±0.04
http	act. rate	0.31±0.07	0.52±0.06	0.73±0.05
ssl	act. rate	0.27±0.06	0.45±0.06	0.68±0.05
files	act. rate	0.18±0.05	0.33±0.05	0.51±0.05
notice	act. rate	0.12±0.04	0.22±0.04	0.36±0.04
proc	act. rate	0.24±0.05	0.41±0.05	0.63±0.05
image	act. rate	0.16±0.04	0.28±0.04	0.46±0.04
svc/reg	act. rate	0.11±0.03	0.21±0.04	0.35±0.04
task	act. rate	0.09±0.03	0.16±0.03	0.27±0.03
socket	act. rate	0.20±0.05	0.36±0.05	0.57±0.05
All sources	avg. MB/episode	38.2±3.6	64.7±4.8	92.4±6.1
All sources	p95 latency [s]	2.4±0.2	2.6±0.2	2.8±0.2

**Table 13:** Ablations at $B_{\mathrm{avg}}=1.3$. Removing any pillar degrades detection or earliness.

Variant	AUROC@B	AUPRC@B	TTD@B	TPR@FPR = 1%
Full model	0.944±0.010	0.915±0.012	18.2±1.8	0.67±0.03
– Generator constraints	0.909±0.013	0.875±0.014	24.8±2.2	0.58±0.03
– Sensor-budget head	0.899±0.014	0.863±0.015	26.2±2.3	0.56±0.04
– Causal CTKG	0.905±0.013	0.871±0.014	25.3±2.2	0.57±0.04

**Table 14:** Robustness evaluation at $B_{\mathrm{avg}}=1.3$.

Condition	AUROC@B	AUPRC@B	TTD@B	p95 Latency [s]
Base	0.944±0.010	0.915±0.012	18.2±1.8	2.6±0.2
Background +30%	0.936±0.011	0.904±0.013	19.1±1.9	2.6±0.2
Missing 20% events	0.927±0.012	0.896±0.013	20.4±2.0	2.6±0.2
Clock skew +250 ms	0.939±0.011	0.907±0.012	19.0±1.9	2.7±0.2
Latency spike p95 + 0.5 s	0.938±0.011	0.905±0.013	19.6±1.9	3.1±0.2

**Table 15:** Counterfactual Preventability $\hat{\mathrm{CP}}\left(S\right)$ at $B_{\mathrm{avg}}=1.3$ for common mid-chain levers. Higher is better.

Technique Set *S*	Description	$\hat{\mathrm{CP}}\left(S\right)$
{T1105}	C2 channel establishment	0.121±0.017
{T1021}	Lateral remote services	0.087±0.015
{T1059,T1105}	Exec → C2 handoff	0.159±0.019
{T1003}	Credential dumping	0.102±0.016

**Table 16:** Explanation Stability XS (Jaccard overlap of important CTKG subgraphs) at matched operating points.

Condition	Within-Card Resample	Across Held-Out Motifs
Sim-CTKG (ours)	0.71±0.04	0.66±0.05
KG-noCausal	0.49±0.05	0.41±0.06
Flat-RL	0.32±0.06	0.28±0.06

**Table 17:** Per-step runtime breakdown (mean ± std) at $B_{\mathrm{avg}}=1.3$ over all cards.

Component	Latency [ms]	Notes
Featurizer	0.7±0.1	rolling sketches and aggregates
Graph encoder	2.3±0.3	$L_{g}=3$, H=4, $d_{\mathrm{hid}}=128$
Policy heads	0.6±0.1	fused MLP projections
Causal update	0.3±0.1	local structural variables
Signing (if emitted)	0.3±0.1	SHA-256 + Ed25519
Total (no artifact)	3.9±0.4	end-to-end step time
Total (with artifact)	4.2±0.4	includes signing

**Table 18:** Provenance manifest size and verification latency.

Artifact	Manifest Size [kB]	Sign Latency [ms]	Verify Latency [ms]
Alert only	6.2±0.5	0.28±0.03	0.41±0.05
Alert + explanation vector	9.8±0.6	0.31±0.04	0.46±0.06
Alert + highlighted subgraph	12.4±0.8	0.34±0.05	0.52±0.06

**Table 19:** Stage-wise median TTD (steps) at $B_{\mathrm{avg}}=1.3$. Lower is better.

Method	Exec (T1059)	C2 (T1105)	Lateral (T1021)	Exfil (T1041)
Sim-CTKG (ours)	12[10,14]	14[12,16]	17[15,20]	19[17,21]
KG-noCausal	16[14,18]	19[17,21]	23[21,26]	26[24,29]
Flat-RL	19[17,22]	22[20,25]	27[24,30]	31[28,34]

**Table 20:** Performance comparison across all baseline methods.

Method	AUROC@B	TTD (Steps)	ECE	Budget Adherence (%)
Fixed-Source (FS)	0.842±0.006	11.3±0.8	0.091±0.012	12.4
Random-Sampling (RS)	0.731±0.010	15.8±1.7	0.142±0.015	48.2
Static Budgeted (SB)	0.801±0.008	13.9±1.1	0.104±0.011	100.0
CTI-only (CTKG-Struct)	0.868±0.009	10.4±0.6	0.076±0.009	100.0
RL w/o CTKG	0.882±0.007	9.7±0.6	0.062±0.010	100.0
RL w/o Cross-Attention	0.891±0.006	9.4±0.5	0.058±0.007	100.0
Sim-CTKG (Ours)	** 0.934±0.004 **	** 7.8±0.4 **	** 0.031±0.006 **	** 100.0 **

**Table 21:** Calibration metrics at $B_{\mathrm{avg}}=1.3$. Lower is better.

Method	ECE	Brier	NLL
Sim-CTKG (ours)	0.028±0.006	0.082±0.008	0.39±0.04
KG-noCausal	0.053±0.009	0.104±0.010	0.51±0.05
Flat-RL	0.071±0.010	0.121±0.011	0.63±0.06

**Table 22:** Seed stability over 10 runs.

Budget	AUROC@B	AUPRC@B	TTD@B	TPR@1% FPR
$B_{\mathrm{avg}}=0.8$	0.915±0.008	0.882±0.010	23.5±1.3	0.61±0.02
$B_{\mathrm{avg}}=1.3$	0.944±0.007	0.915±0.009	18.2±1.1	0.67±0.02
$B_{\mathrm{avg}}=2.0$	0.958±0.006	0.931±0.008	15.4±1.0	0.72±0.02

**Table 23:** Throughput parity at $B_{\mathrm{avg}}=1.3$. FLOPs are approximate per-step forward ops.

Method	Params [M]	FLOPs/Step [M]	Latency/Step [ms]
Sim-CTKG (ours)	7.8	62	3.9±0.4
KG-noCausal	6.2	49	3.2±0.3
Flat-RL	5.1	36	2.7±0.3

**Table 24:** Pairwise tests at $B_{\mathrm{avg}}=1.3$ (stratified by card).

Comparison	AUROC@B *p*	TTD@B *p*	Cohen’s *d* (AUROC)	Cohen’s *d* (TTD)
Ours vs. Flat-RL	<0.001	<0.001	1.12	1.28
Ours vs. KG-noCausal	<0.001	<0.001	0.94	1.03
KG-noCausal vs. Flat-RL	0.004	0.006	0.52	0.61

**Table 25:** Core component ablations at $B_{\mathrm{avg}}=1.3$. AUROC/AUPRC are at budget.

Variant	AUROC@B	AUPRC@B	TTD@B	TPR@1%FPR	ECE
Full model (ours)	0.944±0.010	0.915±0.012	18.2±1.8	0.67±0.03	0.028±0.006
>Generator constraints	0.909±0.013	0.875±0.014	24.8±2.2	0.58±0.03	0.041±0.007
>Sensor-budget head	0.899±0.014	0.863±0.015	26.2±2.3	0.56±0.04	0.046±0.008
>Causal CTKG	0.905±0.013	0.871±0.014	25.3±2.2	0.57±0.04	0.043±0.008
Cross-attn → Concat	0.914±0.012	0.886±0.013	22.1±2.0	0.61±0.03	0.036±0.007
Cross-attn → FiLM	0.923±0.011	0.896±0.013	20.7±2.0	0.63±0.03	0.033±0.007

**Table 26:** Sensitivity to CTKG scope and encoder depth at $B_{\mathrm{avg}}=1.3$. Latency is per step forward time for the graph encoder.

Config $\left(r,L_{g},H\right)$	AUROC@B	AUPRC@B	TTD@B	Graph Latency [ms]
(1, 2, 2)	0.926±0.011	0.893±0.013	20.9±1.9	1.4±0.2
(2, 3, 4) (default)	0.944±0.010	0.915±0.012	18.2±1.8	2.3±0.3
(3, 4, 6)	0.949±0.010	0.919±0.011	17.7±1.8	3.5±0.4
(4, 4, 8)	0.951±0.010	0.921±0.011	17.6±1.8	4.8±0.5

**Table 27:** Budget sweep. Mean ± 95% CI across cards.

Method	$B_{\mathrm{avg}}$	AUROC@B	TTD@B	TPR@1%FPR
Full model (ours)	0.8	0.915±0.011	23.5±2.0	0.61±0.03
	1.3	0.944±0.010	18.2±1.8	0.67±0.03
	2.0	0.958±0.008	15.4±1.5	0.72±0.03
>Sensor-budget head	0.8	0.883±0.014	28.9±2.5	0.53±0.04
	1.3	0.899±0.014	26.2±2.3	0.56±0.04
	2.0	0.914±0.012	23.8±2.2	0.60±0.03
>Causal CTKG	0.8	0.894±0.013	27.6±2.4	0.55±0.04
	1.3	0.905±0.013	25.3±2.2	0.57±0.04
	2.0	0.919±0.012	22.9±2.1	0.61±0.03

**Table 28:** Non-RL detectors at $B_{\mathrm{avg}}=1.3$.

Method	AUROC@B	AUPRC@B	TTD@B	ECE
Sim-CTKG (ours)	0.944±0.010	0.915±0.012	18.2±1.8	0.028±0.006
T-Transformer ($x_{t}$ only)	0.902±0.012	0.855±0.014	24.3±2.1	0.051±0.008
TCN-Detector ($x_{t}$ only)	0.887±0.013	0.838±0.015	26.0±2.3	0.058±0.009
RelGAT-only ($G_{t}$ only)	0.868±0.014	0.814±0.016	27.8±2.4	0.063±0.010
MoE-Selector + Static heads	0.891±0.013	0.844±0.015	25.6±2.2	0.055±0.008

**Table 29:** RL baselines at $B_{\mathrm{avg}}=1.3$.

Method	AUROC@B	AUPRC@B	TTD@B	TPR@1%FPR
Sim-CTKG (ours)	0.944±0.010	0.915±0.012	18.2±1.8	0.67±0.03
Flat-RL ($x_{t}$)	0.846±0.015	0.781±0.017	31.3±2.7	0.47±0.04
KG-noCausal	0.886±0.014	0.829±0.016	27.0±2.3	0.54±0.04
InfoBottleneck-RL	0.904±0.013	0.851±0.015	24.9±2.2	0.57±0.04
Heuristic-RL (selector)	0.878±0.014	0.822±0.016	28.3±2.4	0.52±0.04

**Table 30:** Robustness at $B_{\mathrm{avg}}=1.3$: relative AUROC drop (negative is worse) and ΔTTD in steps.

Condition	Ours	KG-noCausal	Flat-RL	T-Transformer
Background +30%	−0.8%, +0.9	−2.4%, +2.6	−3.6%, +3.8	−3.1%, +3.2
Missing 20% events	−1.8%, +2.2	−4.3%, +3.9	−5.7%, +5.4	−4.9%, +4.8
Clock skew +250 ms	−0.5%, +0.8	−1.6%, +1.9	−2.1%, +2.3	−1.9%, +2.0
Latency spike p95 + 0.5 s	−0.6%, +1.4	−2.0%, +2.7	−3.0%, +3.5	−2.6%, +3.0

**Table 31:** Compute parity at $B_{\mathrm{avg}}=1.3$. FLOPs are approximate forward ops per step.

Method	Params [M]	FLOPs/Step [M]	Latency/Step [ms]
Sim-CTKG (ours)	7.8	62	3.9±0.4
KG-noCausal	6.2	49	3.2±0.3
T-Transformer	8.1	58	3.5±0.3
Flat-RL	5.1	36	2.7±0.3
RelGAT-only	4.6	31	2.4±0.3

## Data Availability

The data that support the findings of this study are available from respective owners of the third-party datasets. Restrictions apply to the availability of these data, which were used under license for this study. The authors of this study donot have permission to distribute the datasets.

## Abbreviations

The following abbreviations are used in this manuscript:

AUC	Area Under Curve
AUROC	Area Under the Receiver Operating Characteristic curve
AUPRC	Area Under the Precision–Recall curve
ATT&CK	Adversarial Tactics, Techniques, and Common Knowledge (MITRE)
BH	Benjamini–Hochberg false discovery rate control
B_avg_	Average sensing cost budget
B_lat_	Latency budget bound (e.g., p95 latency)
CI	Confidence interval
C2	Command and Control
CAPEC	Common Attack Pattern Enumeration and Classification
CP	Counterfactual Preventability
CPE	Common Platform Enumeration
CTI	Cyber Threat Intelligence
CTKG	Cyber-Threat Knowledge Graph
CV	Cross-validation
CVE	Common Vulnerabilities and Exposures
DARPA TCAD	DARPA Transparent Computing attack traces corpus
ECE	Expected Calibration Error
FLOPs	Floating point operations
FPR	False Positive Rate
GAT	Graph Attention Network
GNN	Graph Neural Network
IDS	Intrusion Detection System
KG	Knowledge Graph
MDP	Markov Decision Process
NLL	Negative Log-Likelihood
PPO	Proximal Policy Optimization
PR	Precision–Recall
p95	95th percentile (e.g., latency)
RL	Reinforcement Learning
ROC	Receiver Operating Characteristic
SOC	Security Operations Center
TTP	Tactics, Techniques, and Procedures
TTD	Time To Detect
TTD@B	TTD measured under budget constraints
TPR	True Positive Rate

TPR@1% FPR	TPR at 1% false positive rate
XS	Explanation Stability
Zeek	Open-source network telemetry framework
AUROC@B	AUROC measured under budget constraints
AUPRC@B	AUPRC measured under budget constraints
T1059, T1105, …	MITRE ATT&CK technique IDs used in scenarios
Sim-CTKG	Simulator-aligned CTKG-guided RL detector (this work)
*r*	CTKG slice hop radius
$L_{g}$	Number of graph encoder layers
*H*	Number of attention heads
$x_{t}$	Compact telemetry feature vector at time *t*
$G_{t}$	Two-hop CTKG slice at time *t*

## Notations

**Symbol**	**Meaning**	**Type/Units**
*States, actions, budgets, costs*		
$s_{t}=\left(x_{t},G_{t}\right)$	Agent state at time *t*	feature vector and CTKG slice
$x_{t}\in R^{d_{x}}$	Compact telemetry features at *t*	real vector
$G_{t}=\left(V_{t},E_{t},R\right)$	Two-hop CTKG slice at *t*	directed multi-relational graph
$a_{t}$	Containment decision at *t*	discrete action
$\ell_{t}\in {\left\{0,1\right\}}^{K}$	Logging mask at *t* over *K* sources	binary vector
$C_{t}=\sum_{k=1}^{K}\ell_{t,k}c_{k}$	Sensing cost at *t*	cost units
$c_{k}$	Unit cost of source *k*	cost units
$B_{\mathrm{avg}}$	Average cost budget	cost units
$B_{\mathrm{lat}}$	Latency budget (p95)	seconds
*T*	Episode length	steps
*E*	Number of episodes	count
*Learning and optimization*		
$\pi_{\theta }\left(a_{t},\ell_{t}|s_{t}\right)$	Policy over actions and logging	distribution
$V_{\phi }\left(s_{t}\right)$	State value function	scalar
θ,ϕ	Trainable parameters of policy and value	tensors
$r_{t}$	Reward at *t*	scalar
$r_{t}=r_{\mathrm{detect}}-\beta \;C_{t}$	Reward with cost penalty	scalar
β	Cost trade off coefficient	scalar
γ	Discount factor	scalar
λ	GAE parameter	scalar
η	Learning rate	scalar
τ	Temperature in gating head	scalar
α	Sparsity penalty for logging head	scalar
*CTKG and encoders*		
G=(V,E,R)	Global cyber threat KG	directed multi-relational graph
*r*	CTKG hop radius for slice $G_{t}$	hops
$L_{g}$	Graph encoder layers	count
*H*	Attention heads	count
*d*	Hidden dimension in encoders	count
$h_{v}$	Node embedding for entity *v*	vector
CrossAttn(·)	Cross attention fusion module	function
σ(·)	Logistic function	function
softmax(·)	Normalized exponential	function
⊙	Elementwise product	operator
${∥\;\cdot \;∥}_{2}$	Euclidean norm	operator

*Metrics and operating points*		
AUROC@B	AUROC under budget constraints	[0,1]
AUPRC@B	AUPRC under budget constraints	[0,1]
TPR, FPR	True and false positive rates	[0,1]
TPR@1% FPR	TPR at 1% FPR	[0,1]
TTD, TTD@B	Time to detect (first correct alert)	steps
ECE	Expected Calibration Error	[0,1]
Brier	Brier score	[0,2]
p95	95th percentile (latency)	seconds
*Scenario and provenance*		
M	Set of held out motifs for zero day tests	set
κ	Scenario card parameters distribution	distribution
1[·]	Indicator function	operator
Sig(·)	Provenance signature function	function

## Appendix A. Hyperparameters and Computational Overhead

**Table A1:** Parameters with the settings.

Component	Setting
Reinforcement Learning (PPO)	
Training Steps	1.2 M environment steps
Optimizer	AdamW $\left(\beta_{1}=0.9,\;\beta_{2}=0.999\right)$
Learning Rate	$3\times 10^{-4}$ (cosine decay, 5 k warmup)
Batch Size	4096 transitions
Minibatch Size	512
PPO Clip Ratio	0.20
GAE Parameter λ	0.95
Discount Factor γ	0.99
Entropy Coefficient	0.01
Value Loss Coefficient	0.50
Gradient Norm Clip	0.5
Epochs per PPO Update	10
Seeds Evaluated	10
CTKG and Causal Engine	
CTKG Slice Radius *r*	2 hops
Max Nodes in Slice	70 nodes
Node Embedding Dimension	256
Relation Types	ATT&CK prerequisite, effect, transition edges
CTKG Update Frequency	Every simulation step
SCM Noise Variables $\xi_{k}$	Independent Gaussian
Causal Function Approximation	MLP (2 layers, 128 units)
Cross-Attention Fusion Module	
Attention Heads	4
Cross-Attention Layers	2
Hidden Dimension	256
Dropout	0.10
Fusion Operator	Residual MLP + LayerNorm
Budget Constraints (Dual Variables)	
Average-Cost Budget $B_{\mathrm{avg}}$	1.3 units
Latency Budget $B_{\mathrm{lat}}$	3.0 s (p95)
Dual Step Size ρ	$5\times 10^{-4}$
Penalty Terms	$-\eta_{c}C\left(\delta_{t}\right)-\eta_{\ell }I\left[\mathrm{lat}>B_{\mathrm{lat}}\right]$
Initialization of $\eta_{c},\eta_{\ell }$	0
Projection Domain	$\eta_{c},\eta_{\ell }\geq 0$
Simulator and Telemetry Settings	
Telemetry Types	Process, network flows, file events, authentication logs
Average Telemetry Cost Model	Source-dependent CPU/byte weights
Latency Model	Empirical distribution per telemetry source
Scenario Cards	38 parameterized templates, motif-holdout split
Attack Chains Sampled	5000 training/2000 test
Hardware and Precision	
GPU Used	NVIDIA RTX A6000
Precision	BF16 training, FP16 inference
Total Trainable Parameters	7.8 M
Training Time per Seed	∼4 h

## References

[1] MITRE ATT&CK. https://attack.mitre.org/.

[2] Sharafaldin I., Lashkari A.H., Ghorbani A.A. (2018). Toward Generating a New Intrusion Detection Dataset and Intrusion Traffic Characterization. Proceedings of the 4th International Conference on Information Systems Security and Privacy (ICISSP 2018).

[3] García S., Grill M., Stiborek J., Zunino A. (2014). An Empirical Comparison of Botnet Detection Methods. Comput. Secur..

[4] Anjum M.M., Iqbal S., Hamelin B. (2021). Analyzing the Usefulness of the DARPA OpTC Dataset in Cyber Threat Detection Research. SACMAT ’21: Proceedings of the 26th ACM Symposium on Access Control Models and Technologies.

[5] Brockman G., Cheung V., Pettersson L., Schneider J., Schulman J., Tang J., Zaremba W. (2016). OpenAI Gym. arXiv.

[6] Red Canary Atomic Red Team—Adversary Emulation Tests. https://atomicredteam.io/.

[7] MITRE CALDERA Adversary Emulation Platform. https://caldera.mitre.org/.

[8] Arp D., Quiring E., Pendlebury F., Warnecke A., Pierazzi F., Wressnegger C., Cavallaro L., Rieck K. Dos and Don’ts of Machine Learning in Computer Security. Proceedings of the USENIX Security Symposium.

[9] Alexander O., Belani R. (2023). Attack Flow: Modeling the Adversary.

[10] CVE Program Common Vulnerabilities and Exposures (CVE). https://www.cve.org/.

[11] NIST Common Platform Enumeration (CPE). https://nvd.nist.gov/products/cpe.

[12] Carrara N., Leurent E., Laroche R., Urvoy T., Maillard O.-A., Pietquin O. Budgeted Reinforcement Learning in Continuous State Space. Proceedings of the NeurIPS 2019.

[13] Coalition for Content Provenance and Authenticity (C2PA). C2PA Technical Specification. https://c2pa.org/specifications.

[14] Sutton R.S., Barto A.G. (2018). Reinforcement Learning: An Introduction.

[15] Perez R., Thomas J., Lee O. Mordor—Pre-Recorded Security Telemetry for Detection Research. https://www.deepwatch.com/glossary/security-telemetry/.

[16] Sinaga K.P., Nair A.S. (2025). Calibration Meets Reality: Making Machine Learning Predictions Trustworthy. arXiv.

[17] Altman E. (1999). Constrained Markov Decision Processes.

[18] Pearl J. (2009). Causality: Models, Reasoning, and Inference.

[19] Peters J., Janzing D., Schölkopf B. (2017). Elements of Causal Inference.

[20] NIST (2015). FIPS PUB 180-4: Secure Hash Standard (SHS). https://csrc.nist.gov/publications/detail/fips/180/4/final.

[21] Josefsson S., Liusvaara I. (2017). Edwards-Curve Digital Signature Algorithm (EdDSA). RFC 8032. https://datatracker.ietf.org/doc/html/rfc8032.

[22] Jones M., Bradley J., Sakimura N. (2015). JSON Web Signature (JWS). RFC 7515. https://www.rfc-editor.org/rfc/rfc7515.html.

[23] in-toto Supply Chain Security Framework. https://in-toto.io/specs/.

[24] Cappos J., Samuel J., Baker S., Hartman J.H. (2008). A Look in the Mirror: Attacks on Package Managers. CCS ’08: Proceedings of the 15th ACM Conference on Computer and Communications Security.

[25] Ptacek T., Newsham T. (1998). Insertion, Evasion, and Denial of Service: Eluding Network Intrusion Detection. https://www.academia.edu/37052943/Insertion_Evasion_and_Denial_of_Service_Eluding_Network_Intrusion_Detection.

[26] Microsoft Sysmon—System Monitor. https://learn.microsoft.com/en-us/windows/win32/sysmon/system-monitor-portal.

[27] Microsoft Windows Event Logging. https://learn.microsoft.com/windows/win32/eventlog/event-logging.

[28] Linux Audit Project *auditd(8)*—Linux Audit Daemon. https://linux.die.net/man/8/auditd.

[29] Splunk Threat Research. Splunk Attack Range. https://github.com/splunk/attack_range.

[30] Mordor Datasets Atomic Red Team Telemetry Collections. https://www.atomicredteam.io/.

[31] SigmaHQ Sigma: Generic Signature Format for SIEM Systems. https://sigmahq.github.io/.

[32] Vizcarra J., Gempei Y., Wang Y., Isohara T., Kurokawa M. Constructing Cybersecurity Knowledge Graphs for Hybrid LLM–Graph Reasoning on Vulnerabilities. Proceedings of the 24th International Semantic Web Conference.

[33] Zhou Z., Xu K. (2024). Knowledge Graph Driven Cybersecurity: A Survey. IEEE Commun. Surv. Tutor..

[34] Kiesling E., Ekelhart A., Kurniawan K., Ekaputra F. The SEPSES Knowledge Graph: An Integrated Resource for Cybersecurity. Proceedings of the 18th International Semantic Web Conference.

[35] D’Amico A., Whitley K. (2008). The Real Work of Computer Network Defense Analysts. VizSEC 2007.

[36] Werlinger R., Hawkey K., Beznosov K. (2009). An Integrated View of Human, Organizational, and Technological Challenges of IT Security Management. Inf. Manage. Comput. Secur..

[37] Buczak A.L., Guven E. (2016). A Survey of Data Mining and Machine Learning Methods for Cyber Security Intrusion Detection. IEEE Commun. Surv. Tutor..

[38] Sommer R., Paxson V. Outside the Closed World: On Using Machine Learning for Network Intrusion Detection. Proceedings of the IEEE Symposium on Security and Privacy.

[39] Wang J., Chen Z., Yu X., Li D., Ni J., Tang L., Gui J., Li Z., Chen H., Yu P.S. Heterogeneous Graph Matching Networks for Unknown Malware Detection. Proceedings of the 28th International Joint Conference on Artificial Intelligence.

[40] Tama B.A., Comuzzi M. (2019). Ensemble Learning for Intrusion Detection Systems: A Review. IEEE Access.

[41] Golovin D., Krause A. (2011). Adaptive Submodularity: Theory and Applications in Active Learning and Stochastic Optimization. J. Artif. Intell. Res..

[42] Dulac-Arnold G., Evans R., van Hasselt H., Sunehag P., Lillicrap T., Hunt J., Mann T., Weber T., Degris T., Coppin B. (2015). Deep Reinforcement Learning in Large Discrete Action Spaces. arXiv.

[43] Elastic Security Mapping Detections to MITRE ATT&CK. https://www.elastic.co/guide/en/security/8.19/rules-coverage.html.

[44] Microsoft Security Map Security Operations to MITRE ATT&CK. https://learn.microsoft.com/en-us/security/operations/.

[45] Sikos L.F. (2018). AI in Cybersecurity.

[46] Barnum S., Sethi A. (2014). The Cyber Threat Intelligence (CTI) Technical Landscape.

[47] Husák M., Komárková J., Bou-Harb E., Čeleda P. (2019). Survey of attack projection, prediction, and forecasting in cyber security. IEEE Commun. Surv. Tutor..

[48] Tammoury M. (2022). Post-Incident Forensics with Cybersecurity Knowledge Graphs. Digit. Investig..

[49] Stampa G., Arias M., Sanchez-Charles D., Muntes-Mulero V., Cabellos A. (2017). A Deep-Reinforcement Learning Approach for Software-Defined Networking Routing Optimization. arXiv.

[50] Mao H., Alizadeh M., Menache I., Kandula S. Resource Management with Deep Reinforcement Learning. Proceedings of the 15th ACM Workshop on Hot Topics in Networks.

[51] Sun R., Zhu Y., Fei J., Chen X. (2023). A Survey on Moving Target Defense: Intelligently Changing Attack Surfaces. Appl. Sci..

[52] Yao Q., Wang Y., Xiong X., Wang P., Li Y. (2023). Adversarial Decision-Making for Moving Target Defense: A Multi-Agent Markov Game and Reinforcement Learning Approach. Entropy.

[53] Klein T., Romano G. (2025). Optimizing Cybersecurity Incident Response via Adaptive Reinforcement Learning. J. Adv. Eng. Technol..

[54] Sambasivan R.R., Fonseca R., Shafer I., Ganger G.R. (2014). So, You Want to Trace Your Distributed System? Key Design Insights from Years of Practical Experience.

[55] Red Teaming Research CybORG—Cyber Operations Research Gym. https://github.com/cyborg/cyborg.

[56] Microsoft CyberBattleSim—A Cybersecurity Gym for RL. https://github.com/microsoft/CyberBattleSim.

[57] Elastic Elastic Common Schema (ECS). https://www.elastic.co/guide/en/ecs/current/index.html.

[58] Jiang D., Wang H., Lu Y. (2023). Mastering the Complex Assembly Task With a Dual-Arm Robot: A Novel Reinforcement Learning Method. IEEE Robot. Autom. Mag..

[59] Kober J., Bagnell J.A., Peters J. (2013). Reinforcement Learning in Robotics: Applications and Real-World Challenges. Int. J. Robot. Res..

[60] Splunk Common Information Model (CIM). https://docs.splunk.com/Documentation/CIM/latest/User/Overview.

[61] Snort Project Snort—Network Intrusion Detection and Prevention. https://www.snort.org/.

[62] VirusTotal YARA—The Pattern Matching Swiss Knife for Malware Researchers. https://yara.readthedocs.io/.

[63] Wachter S., Mittelstadt B., Russell C. (2018). Counterfactual Explanations without Opening the Black Box: Automated Decisions and the GDPR. Harv. J. Law Technol..

[64] Karimi A.-H., Barthe G., Balle B., Valera I. Model-Agnostic Counterfactual Explanations for Consequential Decisions. Proceedings of the 23rd International Conference on Artificial Intelligence and Statistics.

[65] Lundberg S.M., Lee S.-I. A Unified Approach to Interpreting Model Predictions. Proceedings of the 31st International Conference on Neural Information Processing System.

[66] Ribeiro M.T., Singh S., Guestrin C. “Why Should I Trust You?”: Explaining the Predictions of Any Classifier. Proceedings of the 22nd ACM SIGKDD International Conference on Knowledge Discovery and Data Mining.

[67] Sigelman B.H., Barroso L.A., Burrows M., Stephenson P., Plakal M., Beaver D., Jaspan S., Shanbhag C. (2010). Dapper, a Large-Scale Distributed Systems Tracing Infrastructure.

[68] OpenTelemetry Sampling Concepts and Guidance. https://opentelemetry.io/docs/concepts/sampling/.

[69] Drewek-Ossowicka A., Pietrołaj M., Rumiński J. (2021). A Survey of Neural Networks Usage for Intrusion Detection Systems. J. Ambient. Intell. Humaniz. Comput..

[70] Abdelhamid S., Aref M., Hegazy I., Roushdy M. A Survey on Learning-BasedIntrusion Detection Systems for IoT Networks. Proceedings of the 2021 Tenth International Conference on Intelligent Computing and Information Systems (ICICIS).

[71] Zeek Project The Book of Zeek—Log Files (conn.log, dns.log, http.log, etc.). https://docs.zeek.org/en/current/script-reference/log-files.html.

[72] Open Information Security Foundation Suricata EVE JSON Output. https://docs.suricata.io/en/latest/output/eve/eve-json-output.html.

[73] Beyer B., Jones C., Petoff J., Murphy N.R. (2016). Site Reliability Engineering: How Google Runs Production Systems.

